# Aberrant activation of the PI3K/mTOR pathway promotes resistance to sorafenib in AML

**DOI:** 10.1038/onc.2016.41

**Published:** 2016-03-21

**Authors:** O Lindblad, E Cordero, A Puissant, L Macaulay, A Ramos, N N Kabir, J Sun, J Vallon-Christersson, K Haraldsson, M T Hemann, Å Borg, F Levander, K Stegmaier, K Pietras, L Rönnstrand, J U Kazi

**Affiliations:** 1 Division of Translational Cancer Research, Department of Laboratory Medicine, Lund University, Lund, Sweden; 2 Lund Stem Cell Center, Department of Laboratory Medicine, Lund University, Lund, Sweden; 3 Department of Hematology and Vascular Disorders, Skåne University Hospital, Lund, Sweden; 4 Department of Pediatric Oncology, Dana-Farber Cancer Institute and Boston Children's Hospital, Harvard Medical School, Boston, MA, USA; 5 Koch Institute for Integrative Cancer Research at Massachusetts Institute of Technology, Massachusetts Institute of Technology, Cambridge, MA, USA; 6 Laboratory of Computational Biochemistry, KN Biomedical Research Institute, Barisal, Bangladesh; 7 Department of Oncology and Pathology, Lund University, Lund, Sweden; 8 Bioinformatics Infrastructure for Life Sciences (BILS), Department of Immunotechnology, Lund University, Lund, Sweden

## Abstract

Therapy directed against oncogenic FLT3 has been shown to induce response in patients with acute myeloid leukemia (AML), but these responses are almost always transient. To address the mechanism of FLT3 inhibitor resistance, we generated two resistant AML cell lines by sustained treatment with the FLT3 inhibitor sorafenib. Parental cell lines carry the FLT3-ITD (tandem duplication) mutation and are highly responsive to FLT3 inhibitors, whereas resistant cell lines display resistance to multiple FLT3 inhibitors. Sanger sequencing and protein mass-spectrometry did not identify any acquired mutations in FLT3 in the resistant cells. Moreover, sorafenib treatment effectively blocked FLT3 activation in resistant cells, whereas it was unable to block colony formation or cell survival, suggesting that the resistant cells are no longer FLT3 dependent. Gene expression analysis of sensitive and resistant cell lines, as well as of blasts from patients with sorafenib-resistant AML, suggested an enrichment of the PI3K/mTOR pathway in the resistant phenotype, which was further supported by next-generation sequencing and phospho-specific-antibody array analysis. Furthermore, a selective PI3K/mTOR inhibitor, gedatolisib, efficiently blocked proliferation, colony and tumor formation, and induced apoptosis in resistant cell lines. Gedatolisib significantly extended survival of mice in a sorafenib-resistant AML patient-derived xenograft model. Taken together, our data suggest that aberrant activation of the PI3K/mTOR pathway in FLT3-ITD-dependent AML results in resistance to drugs targeting FLT3.

## Introduction

Acute myeloid leukemia (AML) is a heterogeneous disease of the blood originating in the bone marrow. Although overall survival of childhood AML has increased in the past decade, it still remains poor compared with that of childhood acute lymphoblastic leukemia. Moreover, survival rates in adults are quite poor and remain virtually unchanged over the last decade.^1^ The molecular genetics of AML has been extensively studied. AML with normal cytogenetics accounts for ~50% of all AML, and this subtype of AML is notable for recurrent mutations in several genes: NPM1, CEBPA, TET2, IDH, DNMT3A and FLT3. The receptor tyrosine kinase FLT3 is expressed at high levels in almost all AML, and >30% of AML bears an oncogenic FLT3 mutation.^2^ The most common FLT3 mutation is an internal tandem duplication (ITD) of the sequence that encodes the juxtamembrane domain, which portends a poor prognosis. Other mutations include point mutations in the kinase domain.

Wild-type FLT3 requires its ligand FL for activation, whereas oncogenic mutants are constitutively active. The key feature of FLT3 activation is phosphorylation of a number of tyrosine residues in the cytoplasmic domain. Phosphotyrosine residues facilitate association with multiple SH2 domain-containing proteins, including cytosolic tyrosine kinases, ubiquitin ligases, adaptor proteins and phosphatases.^3^ Interacting proteins either potentiate receptor signaling by activating multiple pathways, including PI3K-AKT, RAS-RAF-ERK and the p38 pathways, or block receptor signaling by destabilizing the receptor through recruitment of ubiquitin ligases. Oncogenic FLT3 displays equal affinity for the interacting proteins, and thus regulates similar signaling pathways as wild-type FLT3, except for potent activation of STAT5 signaling by FLT3-ITD.^4^

Clinically, FLT3-ITD mutations frequently occur in AML with normal karyotype, t (6:9), t (15:17), and trisomy 8.^5, 6^ The presence of FLT3-ITD does not appear to affect the complete remission rates, but it significantly increases the risk of relapse.^7^ Therefore, expression of FLT3-ITD limits disease-free and overall survival.^8^ FLT3-ITD mutations occur in frame with duplications of 3–400 base pairs in the juxtamembrane domain, and the length of the ITD correlates with overall survival.^9^ Thus, inhibition of FLT3 should be beneficial for patients with AML with constitutively active FLT3 mutants. To date, >20 small molecule FLT3 inhibitors have been developed, 8 of which have been evaluated in clinical trials.^10^ These inhibitors compete with ATP and can efficiently block FLT3 activation as well as downstream signaling. However, none of them has displayed a convincing advancement in AML treatment as a single drug.^10^ Responses were mostly limited to transient reductions in peripheral blood blasts, and bone marrow responses were very rare.^11, 12^ Limited response to the FLT3 inhibitors could be due to several reasons. First, it is possible that FLT3 is efficiently inhibited in cell and animal models by these inhibitors but not in AML in human patients. The use of plasma inhibitory activity assays have addressed this question.^13^ It is also possible that inhibition of FLT3 alone is not sufficient to achieve complete remissions. Another possibility is that primary and secondary mutations in FLT3 make the receptor resistant to these inhibitors.^14^ Earlier studies suggested that acquired mutations in the second part of the kinase domain resulted in a resistant phenotype.^15^ Expression of several survival genes in resistant cells also led to FLT3 inhibitor resistance.^16^ Recently, a second-generation FLT3 inhibitor, AC220 (quizartinib), has been used in a phase II clinical trial for patients with relapsed and chemotherapy-refractory AML and induced a composite complete remission rate of 44–54%. Response was much better than that observed with any other prior FLT3 inhibitor. Later studies suggest, however, that this drug also suffers from secondary resistance.^17^ Another study suggest that the multi-kinase inhibitor midostaurin prolongs survival when used in combination with chemotherapy.^18^ Bone marrow blasts from eight patients with AML treated with quizartinib, who achieved a complete remission and then later relapsed, were studied. All eight were found to have new mutations in the kinase domain of FLT3-ITD.^19^ Thus, the discovery of novel drugs targeting FLT3 or FLT3 downstream or parallel pathways will be useful.

The PI3K-mTOR signaling pathway has been studied extensively in human disease. This pathway has key functions in regulating cell growth, survival and metabolism, and is aberrantly activated in a number of malignant or non-malignant diseases. Components of this pathway have become attractive drug targets and several drugs are in pre-clinical studies or in clinical trials.^20^ The deregulation of the PI3K/mTOR signaling cascade can be specific to the signaling pathway or a consequence of mutations in other pathways that can activate the PI3K/mTOR pathway aberrantly. For instance activating mutations in FLT3, NRAS, KRAS, KIT, the regulatory subunit of PI3K, or loss-of-function mutations of PTEN, can affect this pathway.^3, 21, 22^ Recently, we have shown that, in addition to mutations in these genes, activation of other downstream kinases, such as SYK and p110, contribute to hematopoietic malignancies by activating the PI3K/mTOR pathway.^23, 24^ Several PI3K/mTOR inhibitors have been used in the treatment of solid tumors and hematological malignancies. The majority of these inhibitors target mTOR, whereas others target AKT and PI3K.^20^ Many of them, however, have displayed limited efficacy due to poor specificity and/or poor solubility or bioavailability. Although the PI3K/mTOR pathway is over-represented in hematopoietic malignancies, and mTORC1 is involved in drug resistance, use of drugs targeting this pathway is still limited in the clinic.^25^ A recent study suggests that treatment with a PI3K or AKT inhibitor leads to a differential apoptotic response in 32D cells transfected with FLT3-ITD compared with those expressing FLT3-TKD.^26^ Activation of the STAT5 pathway is partially responsible for the differential effects as FLT3-ITD, but not FLT3-TKD, is a potent activator of this pathway.^4^

In this study, we propose a novel mechanism of FLT3 inhibitor resistance. Using patient-derived cell lines we show that sustained treatment with sorafenib abrogates FLT3 dependency, even though the inhibitor still effectively blocks FLT3 activation in the resistant cells. Exome sequencing of the resistant cell lines revealed that resistant cell lines acquired novel mutations in different signaling proteins and transcription factors. Gene expression and phospho-specific antibody array experiments revealed an enrichment of the PI3K/mTOR signaling pathway in resistant cells. Furthermore, resistant cells responded to PI3K/mTOR inhibitors in cell lines and in mouse xenograft models of AML.

## Results

### Sorafenib-resistant MV4-11 and MOLM-13 cell lines display resistance to AC220

**Figure 1:**
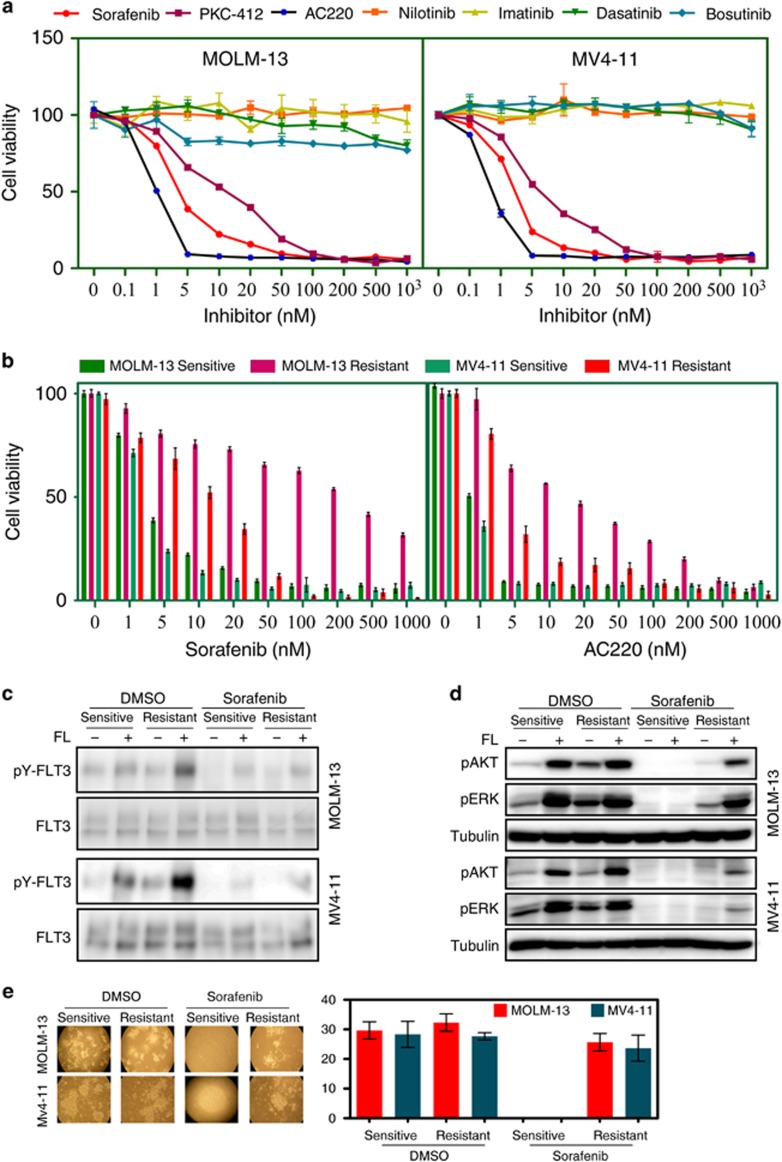
Sorafenib-resistant MV4-11 and MOLM-13 cell lines display resistance to multiple FLT3 inhibitors. (**a**) MOLM-13 and MV4-11 cell lines were treated with an increasing concentration (from 0 to 1000 nM) of multiple tyrosine kinase inhibitors. Cells were cultured with inhibitors for 46 h followed by PrestoBlue viability analysis. (**b**) Sorafenib-sensitive and -resistant cell lines were treated with increasing concentrations of AC220 and sorafenib for 46 h before processing for PrestoBlue viability assays. (**c**) Sorafenib-sensitive and -resistant MOLM-13 and MV4-11 cells were serum-starved for 4 h in the presence or absence of 100 nM sorafenib before 100 ng/ml FL stimulation for 5 min. Cells were then lysed and immunoprecipitated with an anti-FLT3 antibody. The 4G10 (anti-phospho-tyrosine) and anti-FLT3 antibodies were used to probe the blots. (**d**) Cell lysates from the experiment described in **c** were resolved by SDS–PAGE and analyzed by western blotting using anti-phospho AKT, anti-phospho ERK and anti-Tubulin antibodies. (**e**) MOLM-13 and MV4-11 cells were seeded with or without 100 nM sorafenib in semisolid medium and cultured for 7 days.

To identify alternative resistance mechanisms to FLT3 inhibitors in the treatment of FLT3-ITD-driven AML,^10^ we used two patient-derived cell lines, MV4-11 and MOLM-13. MV4-11 cells express only the FLT3-ITD, whereas MOLM-13 cells express wild-type FLT3 and FLT3-ITD. Both cell lines are dependent on FLT3 activity as sorafenib, PKC-412, and AC220, but not imatinib, dasatinib, nilotinib or bosutinib, inhibit cell survival in both cell lines (Figure 1a). After treatment of these cell lines with sorafenib for 90 days, we observed that both cell lines displayed resistance to sorafenib as well as to AC220 (Figure 1b) suggesting that sustained treatment with an FLT3 inhibitor results in acquired resistance. To test whether sorafenib was still effective in FLT3 inhibition, we treated sensitive and resistant cells with sorafenib or dimethyl sulfoxide and then stimulated with FLT3 ligand (FL). Although dimethyl sulfoxide-treated cells responded to FL as expected, sorafenib-treated cells displayed poor FLT3 activation (Figure 1c), suggesting that sorafenib is still capable of inhibiting FLT3 activation in these resistant cells. Surprisingly, we observed that the resistant cells treated with dimethyl sulfoxide had a much more robust response to ligand in terms of FLT3 activation. Similar results were observed with AKT and ERK activation, as sorafenib-treated cells poorly respond to FL stimulation (Figure 1d). Furthermore, resistant cells treated with sorafenib could still form colonies similar to dimethyl sulfoxide-treated cells (Figure 1e), indicating that these cells were no longer dependent on FLT3 activity, although sorafenib could partially block FLT3 activity. Although both MV4-11- and MOLM-13-resistant cell lines displayed similar patterns of FLT3 activity and downstream signaling, MV4-11-resistant cells were more sensitive to sorafenib than MOLM-13-resistant cells, suggesting a complex mechanism behind the resistant phenotype. To determine whether secondary mutations occurred in FLT3, we sequenced the whole coding region using Sanger sequencing. Except for a mutation in the extracellular domain, which was present in all four cell lines (sensitive as well as resistant), we were unable to detect any mutations in the inhibitor-binding site (data not shown). Similar to Sanger sequencing, mass-spectrometric analysis of affinity-enriched FLT3 indicated no differences in FLT3 among the sensitive versus resistant cells in the intracellular part of FLT3 (Supplementary Figure S1). Because we observed an unexpected activation of FLT3 in resistant cells stimulated with FL (Figure 1c), we hypothesized that certain FLT3 residues remain hyper-tyrosine phosphorylated. To test our hypothesis, we used phospho-specific antibodies against known FLT3 residues. Although we observed an increase in total FLT3 phosphorylation, we were unable to identify a single site that was selectively hyper-phosphorylated. Instead, all sites remained slightly more phosphorylated (Supplementary Figure S2) compared with control cells. Therefore, we suggest that sustained treatment with a FLT3 inhibitor abolished FLT3 dependency of cells for survival without the occurrence of any additional secondary mutations in FLT3.

### Resistant cells carry novel mutations compared with sensitive cells

**Figure 2:**
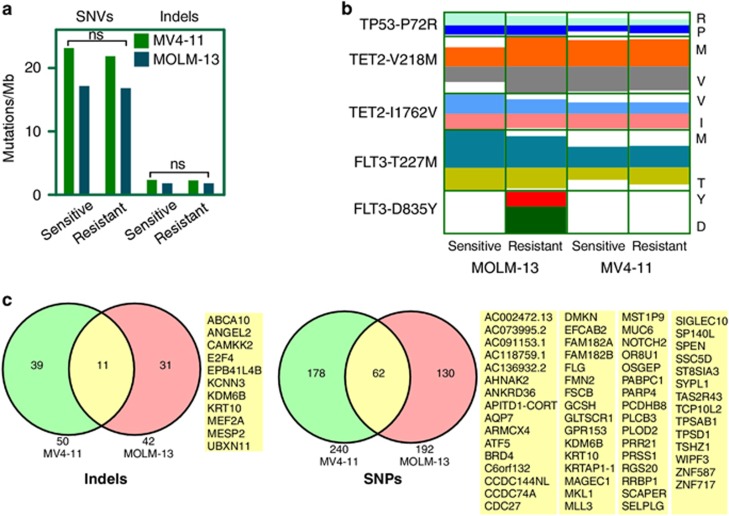
Sorafenib-resistant cell lines carry novel mutations. Genomic DNA from FLT3 inhibitor-sensitive and resistant MV4-11 and MOLM-13 cell lines was extracted using standard protocols. DNA was processed for exome sequencing. (**a**) Comparison of total SNVs per mega bases DNA. (**b**) Known mutations identified in different cell lines. Color code indicates observed allele depth. (**c**) Indels and point mutations were identified by comparing the generated sequence with that from the human reference genome. Genes with novel (not present in sorafenib-sensitive cells) indels and point mutations in both sorafenib-resistant MOLM-13 and MV4-11 cells are reported.

As we were unable to identify any secondary mutation in the inhibitor-binding site of FLT3, we hypothesized that acquired resistance could be due to mutations in genes encoding parallel signaling proteins, leading to hyperactivation of the PI3K/mTOR pathway. Therefore, we sequenced the whole coding region of all genes using a next-generation sequencing approach. FLT3 inhibitor-sensitive and -resistant MV4-11 and MOLM-13 cells were sequenced with an average of a 60-fold coverage and >92% of bases were covered with at least 10 reads. We observed a similar mutational burden in sensitive as well as in resistant cell lines (Figure 2a). In previously reported next-generation sequencing of primary AML samples, recurrent mutations in >20 different genes were demonstrated.^27^ In this subset of genes, we observed mutations in TP53 (P72R), TET2 (V218M and I1762V) and FLT3 (T227M) in all four cell lines (Figure 2b). The FLT3-D835Y mutation was identified in only the MOLM-13-resistant cell line with an allele depth of 67:37, suggesting that the FLT3-D835Y mutation is subclonal, explaining the fact that Sanger sequencing was unable to identify this mutation in FLT3. Probably the FLT3-D835Y mutation also partially contributed to the stronger resistant phenotype observed in MOLM-13 cells (Figures 1b–e) as FLT3-D835Y can render drug resistance. We also observed many novel mutational events in the resistant cell lines compared with the sensitive cells. In MV4-11-resistant cells 52 novel indels (Supplementary Table S1) and 336 novel point mutations (other than synonymous mutations) (Supplementary Table S2) were identified in 50 and 240 genes, respectively. Similar to resistant MV4-11 cells, MOLM-13-resistant cells carried 44 novel indels (Supplementary Table S3) in 42 genes and 279 novel point mutations (Supplementary Table S4) in 192 genes. Novel mutations in 73 genes were common in both resistant cell lines (Figure 2c). According to TCGA data and the Cosmic database, 51 out of 73 genes were found to be mutated in at least one patient. Mutations occurred in several transcriptional regulators, such as E2F4, NOTCH2, ATF5, MKL1, MESP2, MEF2A, MLL3, SPEN, TSHZ1, ZNF587, ZNF717 and KRTAP1-1, and cell surface receptor signaling proteins, such as GPR153, GAB2, NOTCH2, MESP2, NPVF, OR8U1, SPEN and TAS2R43. Furthermore, we observed novel mutations in PIK3C2G (MOLM-13 sorafenib-resistant cells, Supplementary Table S3) and mTOR (MV4-11 sorafenib-resistant cells, Supplementary Table S4) genes in each set of cell lines. The frame-shift mutation observed in the regulatory domain of PI3K has not been reported before. Although several activating mTOR mutations have been described, MV4-11 cells carry E2536A and this mutation has not been previously reported.^28, 29^ Although the finding of mutations in genes involved in the PI3K/mTOR pathway is intriguing, follow-up studies will be needed to determine whether these or the other mutations identified actually contribute to drug resistance.

### PI3K/mTOR pathways are upregulated in resistant cell lines

**Figure 3:**
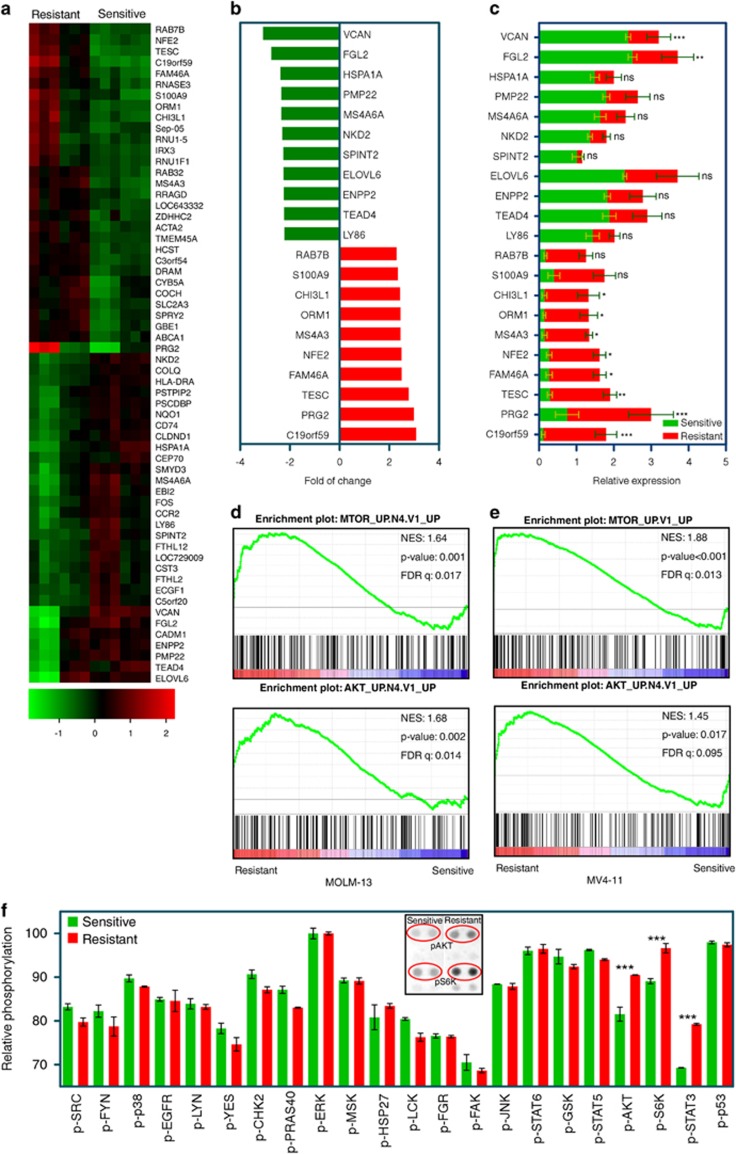
Gene expression profiling suggests an enrichment of the PI3K/mTOR pathways in sorafenib-resistant cells. (**a**) Heatmap of upregulated and downregulated genes in sorafenib-sensitive versus -resistant cells. (**b**) Upregulated and downregulated genes in sorafenib-resistant cells compared with sensitive cells. (**c**) Significantly upregulated and downregulated genes in resistant cells. ANOVA was used to measure the significance. ***P<0.001; **P<0.01; *P<0.05 and ns, *P*>0.05. (**d**, **e**) GSEA was performed using MOLM-13-sensitive cells and resistant cells. GSEA was applied to compare pathways significantly enriched between sorafenib-sensitive and -resistant MOLM-13 (**d**) and MV4-11 (**e**) cells. (**f**) Sorafenib-sensitive and -resistant MOLM-13 cells were serum-starved 4 h before lysis. Lysates were the processed for phospho-specific protein array using manufacturer's protocol. Spots intensities were measured using ImageJ. ***P<0.001. Total phosphorylation was normalized against a loading control.

We next attempted to identify gene expression profiles of resistant cell lines. We analyzed mRNA expression of all cell lines using Illumina HumanHT-12 v4 Expression BeadChips that provides coverage of >47 000 transcripts. We observed that a group of genes responsible for cell survival and proliferation were upregulated in resistant cells, whereas expression of pro-apoptotic genes was downregulated (Figure 3a). To enable prioritization of candidate genes, we analyzed the expression data using ANOVA (Figure 3b) and significance analysis of microarrays (Figure 3c). This analysis further showed that genes previously known to be associated with cell proliferation or cell cycle progression, such as TESC,^30^ FAM46A,^31^ NFE2^32^ and MS4A3^33^ were significantly upregulated. Because gene expression is regulated by sustained expression of signaling cascades, we examined the enrichment of signaling pathways in resistant cell lines. We observed an enrichment of the mTOR and AKT pathways in both MOLM-13- (Figure 3d) and MV4-11- (Figure 3e) resistant cell lines. In addition to pathway enrichment, using a phospho-protein antibody array, we showed that the phosphorylation of the mTOR substrates S6K and AKT were selectively increased in resistant cells (Figure 3f). We also observed an increase in STAT3 phosphorylation. Elevated STAT3 phosphorylation was probably due to the previously described upregulation of JAK3 expression in sorafenib-resistant AML.^34^ Thus, we suggest that the activation of the PI3K/mTOR pathway leads to aberrant expression of survival proteins that further contributes resistance to multiple FLT3 inhibitors in MV4-11- and MOLM-13-resistant cell lines.

### PI3K/mTOR inhibitors are equally effective in inhibiting growth of sensitive and resistant cell lines

**Figure 4:**
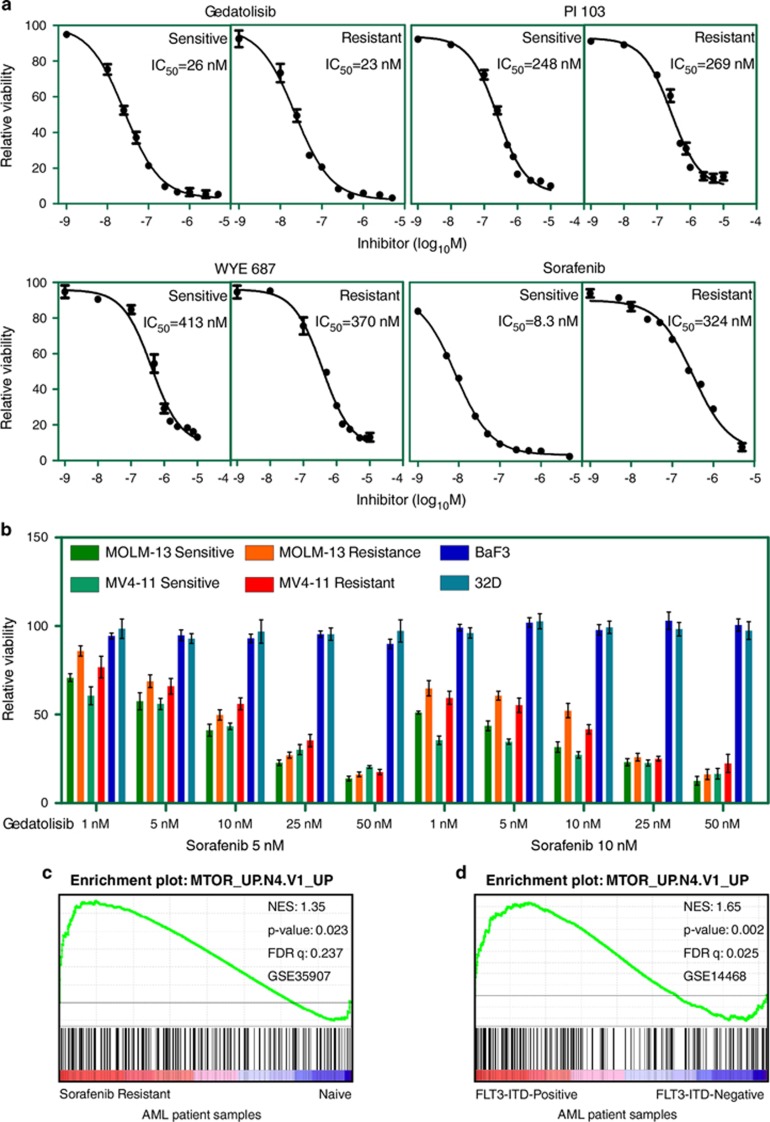
PI3K/mTOR inhibitor effectively reduces cell viability. (**a**) Sorafenib-sensitive and -resistant MOLM-13 cells were treated with different concentrations of inhibitors for 46 h. Cell viability was measured using PrestoBlue cell viability assay. IC_50_ was calculated using GraphPad prism 5.0. (**b**) Sorafenib-sensitive and -resistant MOLM-13 and MV4-11 cells were treated with 5 or 10 nM sorafenib along with increasing concentrations of gedatolisib. Ba/F3 and 32D cells were used as control. (**c**) GSEA was performed using gene expression data from AML patient samples carrying a FLT3-ITD mutation before and after sorafenib treatment. The data set GSE35907 was used for analysis. (**d**) GSEA was performed using gene expression data from AML patient samples carrying FLT3-ITD mutations or not. The data set GSE14468 was used for analysis.

Because we observed that the PI3K/mTOR pathway is upregulated in resistant cells, we treated cells with three PI3K/mTOR inhibitors: gedatolisib (also known as PF 05212384 or PKI-587),^35^ PI 103^36^ and WYE 687.^37^ Although all three inhibitors could inhibit cell viability, gedatolisib, a dual PI3K/mTOR inhibitor that has been studied in solid tumors,^35, 38^ was the most potent inhibitor (IC_50_ 23 nM) in the resistant cell lines (Figure 4a). In addition, we observed that addition of 5 or 10 nM sorafenib in combination with gedatolisib potentiated inhibition of the growth of sorafenib-sensitive cells, but it did not potentiate the effect of gedatolisib on sorafenib-resistant cells (Figure 4b). We also observed enrichment of an mTOR signature in primary blasts from eight samples from patients with sorafenib-resistant AML (GSE35907) (Figure 4c) and in primary patients AML blasts expressing an FLT3-ITD compared with those lacking the FLT3-ITD (525 AML samples, GSE14468) (Figure 4d). Taken together, these data suggest that aberrant activation of the PI3K/mTOR pathway can lead to acquired resistance to sorafenib in AML cells.

### Gedatolisib is a specific PI3K/mTOR inhibitor in AML

**Figure 5:**
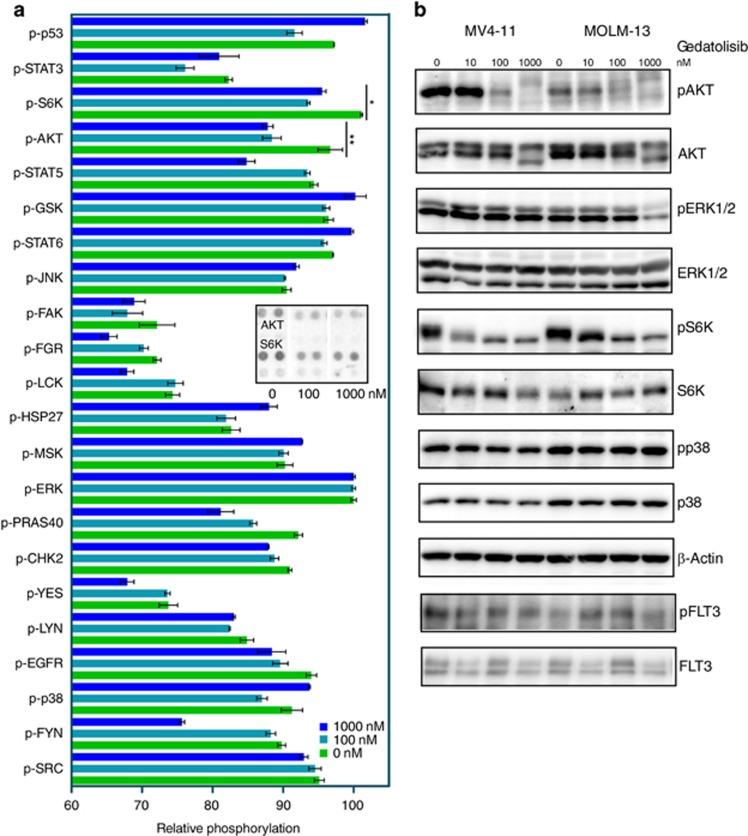
Gedatolisib is a selective PI3K/mTOR inhibitor in AML cell lines. (**a**) Sorafenib-resistant MOLM-13 cells were serum-starved and treated with 0, 100 and 1000 nM gedatolisib for 4 h before lysis. Lysates were then processed for phospho-specific protein array using the manufacturer's protocol. Spot intensities were measured using ImageJ. Total phosphorylation was normalized against a loading control. (**b**) Sorafenib-resistant MV4-11 and MOLM-13 cells were serum-starved and treated with increasing concentration of gedatolisib before lysis. Lysates were then analyzed by western blotting using anti-phospho-specific antibodies. ANOVA was used to measure the significance. **P<0.01; *P<0.05 and ns, *P*>0.05.

Gedatolisib has been shown to be a highly selective inhibitor of PI3K/mTOR.^35^ The inhibitor has been used in cell models, animal models and clinical trials for solid tumors.^38, 39, 40^ To test the specificity of gedatolisib in our cell line model, we ran a phospho-specific antibody array. Treatment of cells with a higher concentration of gedatolisib did not alter the phosphorylation of other signaling proteins, except for AKT and S6K (Figure 5a). The array data were further verified with western blotting using phospho-specific antibodies against AKT, ERK1/2, p38 and S6K (Figure 5b). These results suggest that gedatolisib efficiently blocks the downstream effectors PI3K/mTOR without affecting other signaling pathways. Thus, we suggest that gedatolisib is a specific PI3K/mTOR inhibitor in AML that can be used to block cell growth.

### Gedatolisib inhibits colony formation, cell proliferation and induces apoptosis

**Figure 6:**
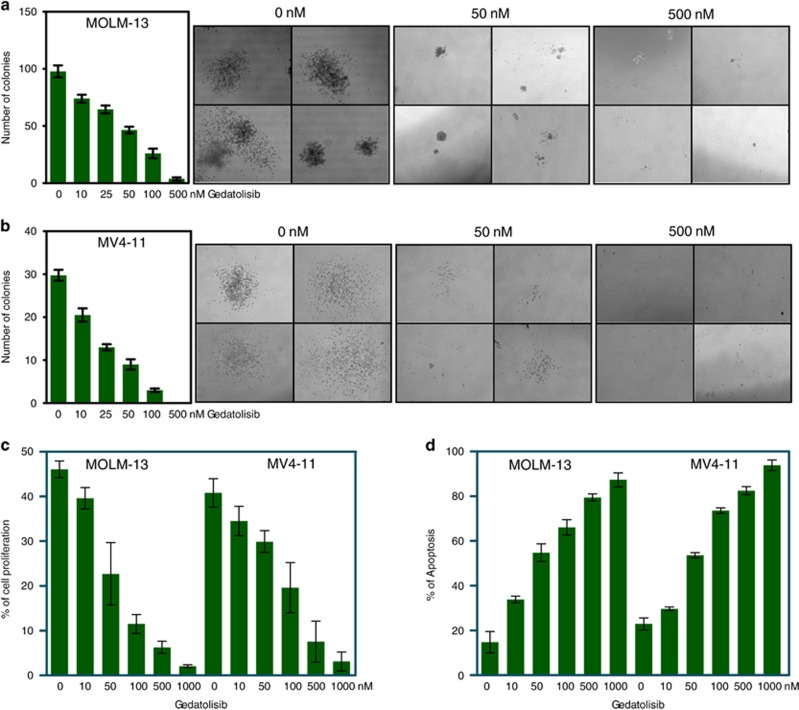
Gedatolisib reduces colony-formation potential, cell proliferation and induces apoptosis. (**a**, **b**) Sorafenib-resistant MOLM-13 (**a**) and MV4-11 (**b**) cells were seeded in semisolid medium containing increasing concentration of gedatolisib. Colonies were counted after 7 days of seeding. (**c**) Sorafenib-resistant cells were seeded with an increasing concentration of inhibitor and incubated for 46 h followed by 2 h of Edu incubation. Cells were then fixed and processed for proliferation assays. (**d**) Sorafenib-resistant cells were seeded with an increasing concentration of inhibitor and incubated for 48 h followed by annexin V and 7-AAD apoptosis assays.

To assess the biological outcomes of gedatolisib treatment, we performed several biological assays including colony formation, cell proliferation and apoptosis studies. Dose-dependent impairment of colony formation suggested that the inhibitor efficiently reduced the number of colonies as well as size of colonies in both MOLM-13- (Figure 6a) and MV4-11- (Figure 6b) resistant cell lines. Furthermore, increasing concentrations of the inhibitor gradually reduced Edu incorporation indicating that the gedatolisib was capable of reducing cell proliferation (Figure 6c). The compound also induced apoptosis in the same cell lines (Figure 6d).

### Gedatolisib delays tumor formation in mouse xenograft

**Figure 7:**
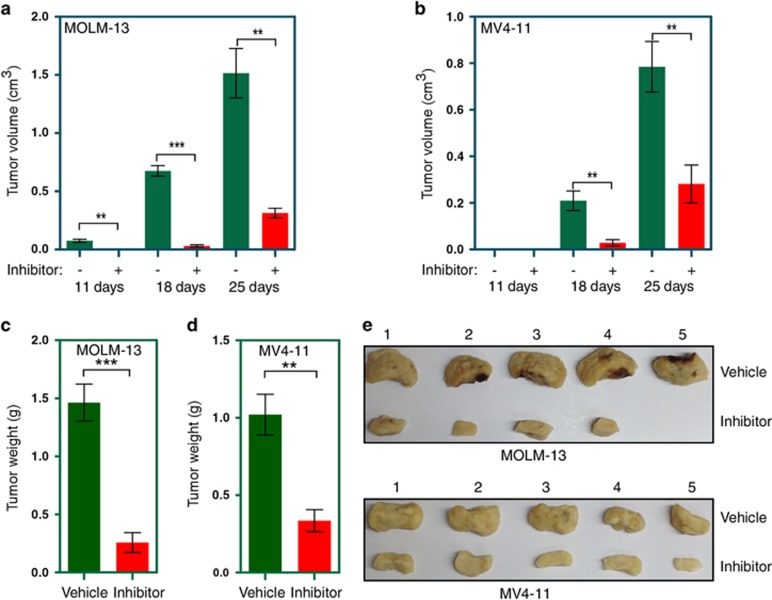
Gedatolisib delays tumor formation in xenograft mice: mice xenografts (five mice in a group) with sorafenib-resistant MOLM-13 (**a**) or MV4-11 (**b**) cells, were treated with gedatolisib or vehicle. Tumor volume was measured at different time points. (**c**–**e**) Tumor weight was measured after dissecting tumor from the inhibitor or vehicle-treated mice.

To test whether gedatolisib is also effective in animal models, we developed mouse AML xenograft models by injecting MV4-11- and MOLM-13-resistant cells subcutaneously. Mice were treated with 12.5 mg/kg of gedatolisib or vehicle for 25 days. As expected, the tumors of vehicle-treated animals grew rapidly. In sharp contrast, tumors developed with a significant delay in mice treated with gedatolisib (Figures 7a and b). Furthermore, gedatolisib treatment significantly reduced tumor weight in both cell lines (Figures 7c–e).

### Gedatolisib significantly extended survival of mice in a sorafenib-resistant patient-derived xenograft model

**Figure 8:**
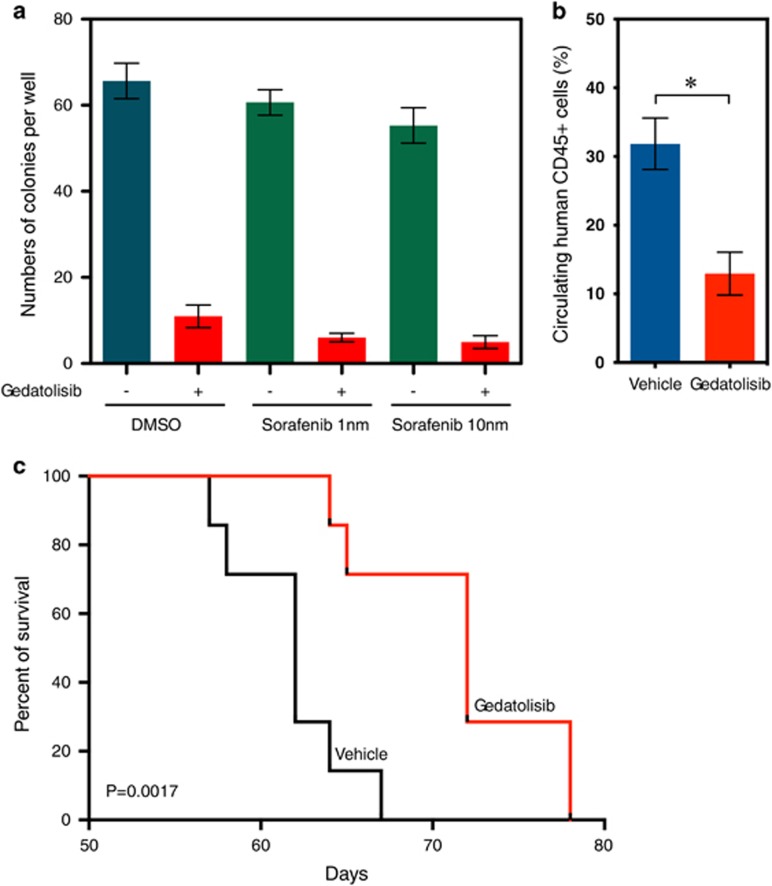
Gedatolisib increases survival of mice in a sorafenib-resistant PDX model. (**a**) Colony-forming units assay from sorafenib-resistant primary AML cells treated with either 1 or 10 nM sorafenib in combination with 50 nM gedatolisib. Results represent the average of triplicate assays. Error bars represent mean±s.e.m. (**b**) Proportion of circulating human CD45-positive AML cells in peripheral blood from four mice per group 58 days post injection. *P*-value calculated using Mann–Whitney test. Error bars represent mean±s.e.m. (**c**) Kaplan–Meier curves showing overall survival of mice (*n*=7 for each group) transplanted with sorafenib-resistant primary cells harvested from a sorafenib-treated patient with AML. Statistical significance determined by log-rank (Mantel–Cox) test. *P-value⩽0.05 by comparison with vehicle-treated group.

To further address the efficacy of gedatolisib in FLT3 inhibitor-resistant AML *in vivo*, we generated a patient-derived xenograft model using an AML sample from a patient with sorafenib-resistant AML. A methylcellulose colony-formation assay demonstrated that gedatolosib effectively reduced colony-formation potential of primary AML cells, and the addition of sorafenib did not significantly increase the inhibitory potential (Figure 8a). Mice treated with gedatolosib displayed a lower number of circulating CD45-positive cells compared with vehicle-treated mice (Figure 8b). Furthermore, gedatolosib significantly extended survival of mice engrafted with sorafenib-resistant AML patient cells (Figure 8c).

## Discussion

Despite ongoing progress in understanding the biological mechanisms of the pathogenesis of AML, patients still suffer from poor outcomes with current therapies. Second-generation FLT3 inhibitors have displayed promising results in some clinical trials. However, development of drug resistance has become a major challenge in targeting this oncogene. In this report, we have shown that aberrant activation of the PI3K/mTOR pathway leads to FLT3 inhibitor resistance in AML, and that a dual PI3K/mTOR pathway inhibitor effectively blocks the growth of these resistant cells *in vitro* and *in vivo*.

Several mechanisms have been described to explain secondary resistance in FLT3-ITD-mutated AML. FLT3-ITD-positive patients harboring D835Y or F961L mutations frequently show reduced response to FLT3 inhibitors.^19, 41^ Moreover, aberrant activation of parallel signaling pathways, such as STAT5 and MAPK, can lead to FLT3 inhibitor resistance even though FLT3 inhibition is maintained.^11^ Our results suggest another mechanism of acquired resistance to sorafenib: activation of the PI3K/mTOR pathway. Hyper-activation of the PI3K/mTOR pathway is known to be involved in leukemia and drug resistance.^25^ The gene expression data suggested an enrichment of the PI3K/mTOR pathway in the resistant cell lines, as well as in primary patient AML blasts, which are resistant to sorafenib therapy. These data were further supported by the phospho-specific-antibody array profiling where we observed that AKT and S6K were selectively activated. Furthermore, an mTOR inhibitor, rapamycin, did not display a growth inhibitory effect (data not shown) suggesting that resistance cells have an upregulation of PI3K/mTOR signaling. The PI3K/mTOR pathway can be activated through several different mechanisms. Amplification or mutations in upstream receptors or signaling proteins frequently activate downstream signaling cascades. With next-generation sequencing we observed novel, non-recurrent mutations in mTOR and PIK3C2G in sorafenib-resistant cell lines and also many overlapping mutations in both cell lines, which might be involved in aberrant activation of the PI3K/mTOR pathway. Consequently, it would be of interest to identify a direct link between the mutations observed in resistant cells and hyperactivation of the PI3K/mTOR pathway. In addition to the alternative pathways, FLT3-D835Y mutation in MOLM-13 cells also contributed a resistant phenotype that was stronger than in the corresponding MV4-11 cells. As FLT3-D835Y occurred only in a small portion of the cells, Sanger sequencing was unable to identify the mutation.

Dual PI3K/mTOR inhibition has been shown to be effective in MLL-rearranged AML cell lines.^42^ In our study, both FLT3 inhibitor-sensitive and resistant human AML cell lines responded to the potent PI3K/mTOR inhibitor gedatolisib.^35^ This is not unexpected because patients carrying FLT3-ITD display upregulation of the PI3K/mTOR signaling pathway. The PI3K/mTOR inhibitor gedatolisib displayed selective target specificity, even at very high concentrations, suggesting that the drug should have few off-target effects. Nano-molar concentrations of the drug induced apoptosis, blocked cell proliferation, abolished colony formation in AML cell lines, delayed tumor formation in a xenograft model, and extended survival in a patient-derived xenograft model, supporting its activity in AML.

In conclusion, we propose an alternative mechanism of FLT3-drug resistance. We show that resistant cells lose FLT3 dependency even though FLT3 remains responsive to the inhibitor. Furthermore, resistant cells display hyperactivation of the PI3K/mTOR pathway and a highly selective inhibitor against this pathway can efficiently block colony formation, decrease cell proliferation, induce apoptosis and block tumor growth *in vivo*.

## Materials and methods

### Patient's data

Patient sample data were collected from two previously published studies. In GSE35907 data set, four AML patient samples carrying FLT3-ITD mutations were treated with sorafenib, and samples were collected before treatment and after developing resistance to the sorafenib.^34^ The data set GSE14468 was generated from a cohort of 598 newly dioagonosed AML patients.^43^

### Cell culture

The human AML cell lines, MV4-11 and MOLM-13 (obtained from DSMZ), were maintained in RPMI-1640 medium (Hyclone, South Logan, Utah) supplemented with 10% heat-inactivated fetal bovine serum (Gibco, Waltham, MA, USA, Australian origin), 100 μg/ml streptomycin and 100 units/ml penicillin. The murine hematopoietic cell line Ba/F3 and the myeloid cell line 32D were cultured in RPMI-1640 medium supplemented with 10% heat-inactivated fetal bovine serum, 10 ng/ml murine interleukin 3, 100 μg/ml streptomycin and 100 units/ml penicillin. Cells were grown at 37 °C in a humidified atmosphere containing 5% CO_2_. All cells were routinely checked for mycoplasma contamination.

### Generation of resistant cell lines

Initially, MV4-11 and MOLM-13 cell lines were treated with sorafenib starting at 1 nM concentration. Complete growth medium with sorafenib was changed twice a week. The concentration of sorafenib was increased to 50 nM within 90 days.

### Antibodies and inhibitors

Anti-FLT3 and anti-phospho-specific FLT3 antibodies were described previously.^44^ Additional antibodies include: anti-phosphotyrosine 4G10 (Millipore, Darmstadt, Germany), anti-phospho AKT (Epitomics, Burlingame, CA, USA), anti-phospho ERK (Santa-Cruz Biotechnology Inc., Dallas, TX, USA), anti-phospho S6K and anti-S6K (Abcam, Cambridge, UK), anti-phospho p38 and anti-p38 (BD biosciences, Sparks, MD, USA) and anti-tubulin and anti-β-actin (Sigma-Aldrich, St Louis, MO, USA). Inhibitors were obtained from TOCRIS chemical (Bristol, UK) and the human phospho-array was from R&D systems (Abingdon, UK).

### Immunoprecipitation and western blotting

After ligand-stimulation and/or inhibitor treatment, cells were washed once with cold phosphate-buffered saline. Cells were then lysed using Triton X-100-based lysis buffer. Cell lysates were mixed with dithiothreitol and sodium dodecyl sulfate polyacrylamide gel electrophoresis containing loading buffer in a 1:1 ratio and boiled before analysis by sodium dodecyl sulfate polyacrylamide gel electrophoresis. For immunoprecipitation, cell lysates were mixed with specific primary antibodies for 1 h on ice followed by protein-A based purification and sodium dodecyl sulfate polyacrylamide gel electrophoresis analysis.

### Cell viability

PrestoBlue cell viability assay (Molecular Probes, Eugene, OR, USA) was used to measure cell viability. Ten thousand cells were seeded per well in 96-well plates in 90 μl medium with or without drugs. Cells were incubated for 46 h. Then 10 μl of PrestoBlue was added to each well. Cells were further incubated for 2 h. Changes of color of PrestoBlue were measured by determining absorbance at 570 and 600 nm. Relative cell viability was calculated as per the manufacturer's instructions.

### Apoptosis

Apoptosis was measured using an annexin V and 7-aminoactinomycin D kit (BD biosciences). Cells positive for annexin V or both annexin V and 7-aminoactinomycin D were counted as apoptotic cells.

### Cell proliferation

Click-iT Edu Alexa Fluor 647 kit was used to measure cell proliferation. Cells were seeded in a 24-well plate with different concentrations of inhibitors and incubated for 46 h. Edu was added to each well and incubated for 2 h before processing cells for staining using the manufacturer's protocols. Cells were then analyzed by flow cytometry.

### Colony-formation assay

Approximately 100 cells were seeded in semisolid methylcellulose medium (Stemcell Technologies, Vancouver, BC, Canada) with different concentrations of inhibitors. Cells were cultured for 7 days before counting colonies.

### Exome sequencing

Total genomic DNA was extracted from cell lines using a DNeasy Blood and Tissue kits (Qiagen, Copenhagen, Denmark). The Human All Exon enrichment (Agilent SureSelectXT) library was used to read 100 bp paired end sequencing on a Genome Sequencer Illumina HiSeq2500. Sequence reads are mapped to the reference sequence (human, hg19, GRCh37) using Burrows-Wheeler Aligner with the default parameters. The SNP and InDel calling is done using Genome Analysis Toolkit's Unified Genotyper, which use Bayesian genotype likelihood model to estimate simultaneously the most likely genotypes and allele frequency in a population of *N* samples.

### Mass spectrometric analysis of FLT3

Immunoprecipitated FLT3 was separated by sodium dodecyl sulfate polyacrylamide gel electrophoresis, reduced and alkylated before separate in-gel digestions using trypsin and LysC, respectively. The digests were separated by nano-LC (Eksigent) and analyzed online by liquid chromatography–mass-spectrometry (LC-MS)/MS on an Orbitrap XL ETD. MS/MS spectra were matched against the human part of Swissprot as of 2013-02, extended with an equal size reverse sequence database using Mascot v 2.4.1(www.matrixscience.com). For MS/MS matching a precursor tolerance of 7 ppm and a fragment tolerance of 0.5 Da were used. A label-free quantification workflow^45^ was used for FLT3 sequence coverage comparisons, and peptides, which were detected in at least one file with a false discovery rate below 1% as determined in the Proteios Software Environment,^46^ were included in the comparison.

### Mouse xenograft model

NOD/SCID mice were injected with 200 000 cells subcutaneously and treated, from the subsequent day after injection, with twice weekly intravenous 12 mg/kg gedatolisib or vehicle (5% (278 mM) dextrose in water, 0.3% lactic acid pH 3.5) for 25 days. Experiment was performed following Swedish animal authority approved protocols.

### Microarray analysis

Total RNA was extracted from cells using RNeasy mini kit (Qiagen). Illumina bead array technology was used to analyze mRNA expression using Illumina HumanHT-12 v4 Expression BeadChip. Gene expression was compared using significant microarray analysis tools and gene set enrichment analysis.

### Patient-derived xenograft model

Blasts from a sorafenib-resistant patient with AML were collected from bone marrow aspirates after obtaining patient informed consent under a Dana-Farber Cancer Institute Internal Review Board-approved protocol. Mononuclear cells were isolated using Ficoll-Paque Plus (Amersham Biosciences, Little Chalfont, UK) and red blood cells were lysed before tail-vein injection into 250 cGy-irradiated NSG (Nod Scid Gamma) mice. After disease burden, human AML blasts were harvested and reinjected into secondary NSG recipients (1.5 × 10^6^ cells per mouse) separated into two groups treated twice a week by tail-vein injection with 100 μl vehicle (5% dextrose in water, 0.3% lactic acid pH 3.5), or gedatolisib (12 mg/kg). Treatment started 3 weeks after cell injection. Disease burden was monitored by evaluation of circulating human CD45+ AML cells in peripheral blood using flow cytometry.

### Colony-formation assay for primary AML cells

Colony–plating assays were performed in methylcellulose-based medium MethoCult GF M3434 (StemCell Technologies). In total, 1 × 10^4^ sorafenib-resistant primary cells were plated in triplicate, treated with either 1 or 10 nM sorafenib in combination with 50 nM gedatolisib, and scored for colony formation 14 days later.

### Statistical analysis

GraphPad Prism was used for statistical analysis. Data were shown as mean value and error bar represents s.e.m. All tests were two-sided.

## References

[bib1] Juliusson G, Antunovic P, Derolf A, Lehmann S, Möllgård L, Stockelberg D et al. Age and acute myeloid leukemia: real world data on decision to treat and outcomes from the Swedish Acute Leukemia Registry. Blood 2009; 113: 4179–4187. 19008455 10.1182/blood-2008-07-172007

[bib2] Gilliland DG, Griffin JD. The roles of FLT3 in hematopoiesis and leukemia. Blood 2002; 100: 1532–1542. 12176867 10.1182/blood-2002-02-0492

[bib3] Masson K, Rönnstrand L. Oncogenic signaling from the hematopoietic growth factor receptors c-Kit and Flt3. Cell Signal 2009; 21: 1717–1726. 19540337 10.1016/j.cellsig.2009.06.002

[bib4] Hayakawa F, Towatari M, Kiyoi H, Tanimoto M, Kitamura T, Saito H et al. Tandem-duplicated Flt3 constitutively activates STAT5 and MAP kinase and introduces autonomous cell growth in IL-3-dependent cell lines. Oncogene 2000; 19: 624–631. 10698507 10.1038/sj.onc.1203354

[bib5] Fröhling S, Schlenk RF, Breitruck J, Benner A, Kreitmeier S, Tobis K et al. Prognostic significance of activating FLT3 mutations in younger adults (16 to 60 years) with acute myeloid leukemia and normal cytogenetics: a study of the AML Study Group Ulm. Blood 2002; 100: 4372–4380. 12393388 10.1182/blood-2002-05-1440

[bib6] Schnittger S, Schoch C, Dugas M, Kern W, Staib P, Wuchter C et al. Analysis of FLT3 length mutations in 1003 patients with acute myeloid leukemia: correlation to cytogenetics, FAB subtype, and prognosis in the AMLCG study and usefulness as a marker for the detection of minimal residual disease. Blood 2002; 100: 59–66. 12070009 10.1182/blood.v100.1.59

[bib7] Kiyoi H, Yanada M, Ozekia K. Clinical significance of FLT3 in leukemia. Int J Hematol 2005; 82: 85–92. 16146837 10.1532/IJH97.05066

[bib8] Sheikhha MH, Awan A, Tobal K, Liu Yin JA. Prognostic significance of FLT3 ITD and D835 mutations in AML patients. Haematol J 2003; 4: 41–46. 10.1038/sj.thj.6200224 12692519

[bib9] Meshinchi S, Stirewalt DL, Alonzo TA, Boggon TJ, Gerbing RB, Rocnik JL et al. Structural and numerical variation of FLT3/ITD in pediatric AML. Blood 2008; 111: 4930–4933. 18305215 10.1182/blood-2008-01-117770 PMC2384125

[bib10] Leung AY, Man CH, Kwong YL. FLT3 inhibition: a moving and evolving target in acute myeloid leukaemia. Leukemia 2013; 27: 260–268. 22797419 10.1038/leu.2012.195

[bib11] Knapper S, Mills KI, Gilkes AF, Austin SJ, Walsh V, Burnett AK. The effects of lestaurtinib (CEP701) and PKC412 on primary AML blasts: the induction of cytotoxicity varies with dependence on FLT3 signaling in both FLT3-mutated and wild-type cases. Blood 2006; 108: 3494–3503. 16868253 10.1182/blood-2006-04-015487

[bib12] Stone RM, DeAngelo DJ, Klimek V, Galinsky I, Estey E, Nimer SD et al. Patients with acute myeloid leukemia and an activating mutation in FLT3 respond to a small-molecule FLT3 tyrosine kinase inhibitor, PKC412. Blood 2005; 105: 54–60. 15345597 10.1182/blood-2004-03-0891

[bib13] Kayser S, Levis MJ. FLT3 tyrosine kinase inhibitors in acute myeloid leukemia: clinical implications and limitations. Leuk Lymphoma 2014; 55: 243–255. 23631653 10.3109/10428194.2013.800198 PMC4333682

[bib14] Breitenbuecher F, Markova B, Kasper S, Carius B, Stauder T, Böhmer FD et al. A novel molecular mechanism of primary resistance to FLT3-kinase inhibitors in AML. Blood 2009; 113: 4063–4073. 19144992 10.1182/blood-2007-11-126664

[bib15] von Bubnoff N, Engh RA, Aberg E, Sanger J, Peschel C, Duyster J. FMS-like tyrosine kinase 3-internal tandem duplication tyrosine kinase inhibitors display a nonoverlapping profile of resistance mutations *in vitro*. Cancer Res 2009; 69: 3032–3041. 19318574 10.1158/0008-5472.CAN-08-2923

[bib16] Stolzel F, Steudel C, Oelschlagel U, Mohr B, Koch S, Ehninger G et al. Mechanisms of resistance against PKC412 in resistant FLT3-ITD positive human acute myeloid leukemia cells. Ann Hematol 2010; 89: 653–662. 20119833 10.1007/s00277-009-0889-1

[bib17] Smith CC, Lasater EA, Zhu X, Lin KC, Stewart WK, Damon LE et al. Activity of ponatinib against clinically-relevant AC220-resistant kinase domain mutants of FLT3-ITD. Blood 2013; 121: 3165–3171. 23430109 10.1182/blood-2012-07-442871 PMC3630831

[bib18] Stone RM, Mandrekar S, Sanford BL, Geyer S, Bloomfield CD, Dohner K et al. The Multi-Kinase Inhibitor Midostaurin (M) Prolongs Survival Compared with Placebo (P) in Combination with Daunorubicin (D)/Cytarabine (C) Induction (ind), High-Dose C Consolidation (consol), and As Maintenance (maint) Therapy in Newly Diagnosed Acute My…. Blood 2015; 126: 6–6.26138538

[bib19] Smith CC, Wang Q, Chin CS, Salerno S, Damon LE, Levis MJ et al. Validation of ITD mutations in FLT3 as a therapeutic target in human acute myeloid leukaemia. Nature 2012; 485: 260–263. 22504184 10.1038/nature11016 PMC3390926

[bib20] Barrett D, Brown VI, Grupp SA, Teachey DT. Targeting the PI3K/AKT/mTOR signaling axis in children with hematologic malignancies. Paediatr Drugs 2012; 14: 299–316. 22845486 10.2165/11594740-000000000-00000 PMC4214862

[bib21] Birkenkamp KU, Geugien M, Schepers H, Westra J, Lemmink HH, Vellenga E. Constitutive NF-kappaB DNA-binding activity in AML is frequently mediated by a Ras/PI3-K/PKB-dependent pathway. Leukemia 2004; 18: 103–112. 14574326 10.1038/sj.leu.2403145

[bib22] Lennartsson J, Rönnstrand L. Stem cell factor receptor/c-Kit: from basic science to clinical implications. Physiol Rev 2012; 92: 1619–1649. 23073628 10.1152/physrev.00046.2011

[bib23] Puissant A, Fenouille N, Alexe G, Pikman Y, Bassil CF, Mehta S et al. SYK is a critical regulator of FLT3 in acute myeloid leukemia. Cancer Cell 2014; 25: 226–242. 24525236 10.1016/j.ccr.2014.01.022 PMC4106711

[bib24] Sun J, Mohlin S, Lundby A, Kazi JU, Hellman U, Påhlman S et al. The PI3-kinase isoform p110delta is essential for cell transformation induced by the D816V mutant of c-Kit in a lipid-kinase-independent manner. Oncogene 2014; 33: 5360–5369. 24213578 10.1038/onc.2013.479

[bib25] Martelli AM, Evangelisti C, Chappell W, Abrams SL, Basecke J, Stivala F et al. Targeting the translational apparatus to improve leukemia therapy: roles of the PI3K/PTEN/Akt/mTOR pathway. Leukemia 2011; 25: 1064–1079. 21436840 10.1038/leu.2011.46

[bib26] Nogami A, Oshikawa G, Okada K, Fukutake S, Umezawa Y, Nagao T et al. FLT3-ITD confers resistance to the PI3K/Akt pathway inhibitors by protecting the mTOR/4EBP1/Mcl-1 pathway through STAT5 activation in acute myeloid leukemia. Oncotarget 2015; 6: 9189–9205. 25826077 10.18632/oncotarget.3279 PMC4496211

[bib27] Cancer Genome Atlas Research N. Genomic and epigenomic landscapes of adult de novo acute myeloid leukemia. N Engl J Med 2013; 368: 2059–2074. 23634996 10.1056/NEJMoa1301689 PMC3767041

[bib28] Grabiner BC, Nardi V, Birsoy K, Possemato R, Shen K, Sinha S et al. A diverse array of cancer-associated MTOR mutations are hyperactivating and can predict rapamycin sensitivity. Cancer Discov 2014; 4: 554–563. 24631838 10.1158/2159-8290.CD-13-0929 PMC4012430

[bib29] Yang H, Rudge DG, Koos JD, Vaidialingam B, Yang HJ, Pavletich NP. mTOR kinase structure, mechanism and regulation. Nature 2013; 497: 217–223. 23636326 10.1038/nature12122 PMC4512754

[bib30] Man CH, Lam SS, Sun MK, Chow HC, Gill H, Kwong YL et al. A novel tescalcin-sodium/hydrogen exchange axis underlying sorafenib resistance in FLT3-ITD+ AML. Blood 2014; 123: 2530–2539. 24608976 10.1182/blood-2013-07-512194

[bib31] Cui J, Wang W, Lai MD, Xu EP, Lv BJ, Lin J et al. Identification of a novel VNTR polymorphism in C6orf37 and its association with colorectal cancer risk in Chinese population. Clin Chim Acta 2006; 368: 155–159. 16545789 10.1016/j.cca.2005.12.043

[bib32] Wang W, Schwemmers S, Hexner EO, Pahl HL. AML1 is overexpressed in patients with myeloproliferative neoplasms and mediates JAK2V617F-independent overexpression of NF-E2. Blood 2010; 116: 254–266. 20339092 10.1182/blood-2009-11-254664 PMC2910609

[bib33] Kutok JL, Yang X, Folkerth R, Adra CN. Characterization of the expression of HTm4 (MS4A3), a cell cycle regulator, in human peripheral blood cells and normal and malignant tissues. J Cell Mol Med 2011; 15: 86–93. 19818099 10.1111/j.1582-4934.2009.00925.x PMC3822496

[bib34] Man CH, Fung TK, Ho C, Han HH, Chow HC, Ma AC et al. Sorafenib treatment of FLT3-ITD(+) acute myeloid leukemia: favorable initial outcome and mechanisms of subsequent nonresponsiveness associated with the emergence of a D835 mutation. Blood 2012; 119: 5133–5143. 22368270 10.1182/blood-2011-06-363960

[bib35] Venkatesan AM, Dehnhardt CM, Delos Santos E, Chen Z, Dos Santos O, Ayral-Kaloustian S et al. Bis(morpholino-1,3,5-triazine) derivatives: potent adenosine 5'-triphosphate competitive phosphatidylinositol-3-kinase/mammalian target of rapamycin inhibitors: discovery of compound 26 (PKI-587), a highly efficacious dual inhibitor. J Med Chem 2010; 53: 2636–2645. 20166697 10.1021/jm901830p

[bib36] Sasore T, Kennedy B. Deciphering combinations of PI3K/AKT/mTOR pathway drugs augmenting anti-angiogenic efficacy *in vivo*. PloS One 2014; 9: e105280. 25144531 10.1371/journal.pone.0105280 PMC4140730

[bib37] Yu K, Toral-Barza L, Shi C, Zhang WG, Lucas J, Shor B et al. Biochemical, cellular, and *in vivo* activity of novel ATP-competitive and selective inhibitors of the mammalian target of rapamycin. Cancer Res 2009; 69: 6232–6240. 19584280 10.1158/0008-5472.CAN-09-0299

[bib38] Mallon R, Feldberg LR, Lucas J, Chaudhary I, Dehnhardt C, Santos ED et al. Antitumor efficacy of PKI-587, a highly potent dual PI3K/mTOR kinase inhibitor. Clinical Cancer Res 2011; 17: 3193–3203. 21325073 10.1158/1078-0432.CCR-10-1694

[bib39] D'Amato V, Rosa R, D'Amato C, Formisano L, Marciano R, Nappi L et al. The dual PI3K/mTOR inhibitor PKI-587 enhances sensitivity to cetuximab in EGFR-resistant human head and neck cancer models. Br J Cancer 2014; 110: 2887–2895. 24823695 10.1038/bjc.2014.241 PMC4056056

[bib40] Gedaly R, Angulo P, Hundley J, Daily MF, Chen C, Evers BM. PKI-587 and sorafenib targeting PI3K/AKT/mTOR and Ras/Raf/MAPK pathways synergistically inhibit HCC cell proliferation. J Surg Resh 2012; 176: 542–548. 10.1016/j.jss.2011.10.045 22261591

[bib41] Albers C, Leischner H, Verbeek M, Yu C, Illert AL, Peschel C et al. The secondary FLT3-ITD F691L mutation induces resistance to AC220 in FLT3-ITD+ AML but retains *in vitro* sensitivity to PKC412 and Sunitinib. Leukemia 2013; 27: 1416–1418. 23392356 10.1038/leu.2013.14

[bib42] Sandhofer N, Metzeler KH, Rothenberg M, Herold T, Tiedt S, Groiss V et al. Dual PI3K/mTOR inhibition shows antileukemic activity in MLL-rearranged acute myeloid leukemia. Leukemia 2015; 29: 828–838. 25322685 10.1038/leu.2014.305

[bib43] Wouters BJ, Löwenberg B, Erpelinck-Verschueren CA, van Putten WL, Valk PJ, Delwel R. Double CEBPA mutations, but not single CEBPA mutations, define a subgroup of acute myeloid leukemia with a distinctive gene expression profile that is uniquely associated with a favorable outcome. Blood 2009; 113: 3088–3091. 19171880 10.1182/blood-2008-09-179895 PMC2662648

[bib44] Razumovskaya E, Masson K, Khan R, Bengtsson S, Rönnstrand L. Oncogenic Flt3 receptors display different specificity and kinetics of autophosphorylation. Exp Hematol 2009; 37: 979–989. 19477218 10.1016/j.exphem.2009.05.008

[bib45] Sandin M, Ali A, Hansson K, Månsson O, Andreasson E, Resjö S et al. An adaptive alignment algorithm for quality-controlled label-free LC-MS. Mol Cell Proteomics 2013; 12: 1407–1420. 23306530 10.1074/mcp.O112.021907 PMC3650348

[bib46] Häkkinen J, Vincic G, Månsson O, Wårell K, Levander F. The proteios software environment: an extensible multiuser platform for management and analysis of proteomics data. J Proteome Res 2009; 8: 3037–3043. 19354269 10.1021/pr900189c

