# Supplementary material for: Aberrant activation of the PI3K/mTOR pathway promotes resistance to sorafenib in AML
Source: Oncogene. 2016 Mar 21;35(39):5119–31. doi: 10.1038/onc.2016.41 (PMC5399143; doi:10.1038/onc.2016.41)

# Fig. S1: FLT3 protein sequence. Peptides matched by LC-MS/MS shown in color

| | | | | | |
|---|---|---|---|---|---|
| 1 | MPALARDGGQ | LPLLVVFSAM | IFGTITNQDL | PVIKCVLINH | KNNDSSVGKS |
| 51 | SSYPMVSESP | EDLGCALRPQ | SSGTVYEAAA | VEVDVSASIT | LQVLVDAPGN |
| 101 | ISCLWVFKHS | SLNCQPHFDL | QNRGVVSMVI | LKMTETQAGE | YLLFIQSEAT |
| 151 | NYTILFTVSI | RNTLLYTLRR | PYFRKMENQD | ALVCISESVP | EPIVEWVLCD |
| 201 | SQGESCKEES | PAVVKKEEKV | LHELFGTDIR | CCARNELGRE | CTRLFTIDLN |
| 251 | QTPQTTLPQL | FLKVGEPLWI | RCKAVHVNHG | FGLTWELENK | ALEEGNYFEM |
| 301 | STYSTNRTMI | RILFAFVSSV | ARNDTGYYTC | SSSKHPSQSA | LVTIVEKGFI |
| 351 | NATNSSEDYE | IDQYEEFCFS | VRFKAYPQIR | CTWTFSRKSF | PCEQKGLDNG |
| 401 | YSISKFCNHK | HQPGEYIFHA | ENDDAQFTKM | FTLNIRRKPQ | VLAEASASQA |
| 451 | SCFSDGYPLP | SWTWKKCSDK | SPNCTEEITE | GVWNRKANRK | VFGQWVSSST |
| 501 | LNMSEAIKGF | LVKCCAYNSL | GTSCETILLN | SPGPFPFIQD | NISFYATIGV |
| 551 | CLLFIVVLTL | LICHKYKKQF | RYESQLQMVQ | VTGSSDNEYF | YVDFREYEYD |
| 601 | LKWEFPRENL | EFGKVLGSGA | FGKVMNATAY | GISKTGVSIQ | VAVKMLKEKA |
| 651 | DSSEREALMS | ELKMMTQLGS | HENIVNLLGA | CTLSGPIYLI | FEYCCYGDLL |
| 701 | NYLRSKREKF | HRTWTEIFKE | HNFSFYPTFQ | SHPNSSMPGS | REVQIHPDSD |
| 751 | QISGLHGNSF | HSEDEIEYEN | QKRLEEEEDL | NVLTFEDLLC | FAYQVAKGME |
| 801 | FLEFKSCVHR | DLAARNVLVT | HGKVVKICDF | GLA**R**DIMSDS | NYVVRGNARL |
| 851 | PVKWMAPESL | FEGIYTIKSD | VWSYGILLWE | IFSLGVNPYP | GIPVDANFYK |
| 901 | LIQNGFKMDQ | PFYATEEIYI | IMQSCWAFDS | RKRPSFPNLT | SFLGCQLADA |
| 951 | EEAMYQNVDG | RVSECPHTYQ | NRRPFSREMD | LGLLSPQAQV | EDS |

Yellow (LysC) /Red (Trypsin)/Dark red (LysC+Trypsin) Detected in all four

Green (Trpsin) Not detected in 11R and 13R

Dark blue (Trypsin) Not detected in 13R

Known phosphosites

Fig. S2: Phosphorylation of different FLT3 phosphorylation sites in MOLM-13 sensitive and resistant cells. MOLM-13 cells were stimulated with 100 ng/ml FL for 0, 5, and 15 minutes. Total cell lysates were analyzed with western blotting using different anti-phospho-specific FLT3 antibodies. Band intensities were measured using ImageJ. Phosphorylation was normalized against total FLT3.

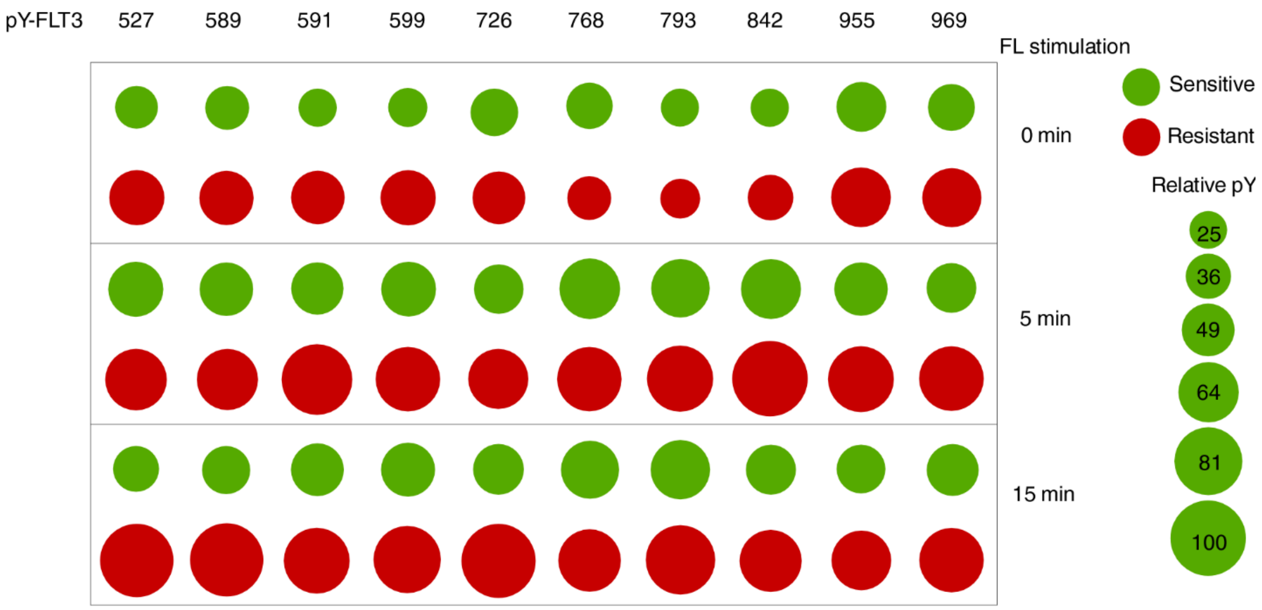

Supplement: Supplementary Figures [file onc201641x1.pdf]
