# Supplementary material for: Aberrant activation of the PI3K/mTOR pathway promotes resistance to sorafenib in AML
Source: Oncogene. 2016 Mar 21;35(39):5119–31. doi: 10.1038/onc.2016.41 (PMC5399143; doi:10.1038/onc.2016.41)
Supplement: Supplementary Tables [file onc201641x2.pdf]

Contents:

Table S1: Novel indels found in genes of MV4-11 resistant cells Page 2

Table S2: Novel indels found in genes of MOLM-13 resistant cells Page 5

Table S3: Novel SNPs found in genes of MV4-11 resistant cells Page 7

Table S4: Novel SNPs found in genes of MOLM-13 resistant cells Page 24

Table S1: Novel indels found in genes of MV4-11 resistant cells

CHR	POSITION	REFERENCE BASE	OBSERVED BASE	QUALITY SCORE	FILTER	COV	EFFECT	GENE NAME	TRANSCRIPT ID
chr17	67160234	ATGGTTTTAT	A	398.54	PASS	15	CODON_CHANGE_PLUS _CODON_DELETION	ABCA10	ENST00000269081
chr8	8176387	C	CGGGGCG	498.8	PASS	13	CODON_INSERTION	AC068353.1	ENST00000330777
chr17	30469470	CCCG	C	57.01	PASS	6	CODON_DELETION	AC090616.2	ENST00000398832
chr1	213180441	GA	G	22	LowQual; QDFilter	13	FRAME_SHIFT	ANGEL2	ENST00000310246
chrX	66766356	TGGCGGCGGCGG CGGC	T	464.33	PASS	7	CODON_DELETION	AR	ENST00000544984
chr6	16327864	GTGC	G,GTGCTGC	1477	PASS	76	CODON_CHANGE_PLUS _CODON_DELETION	ATXN1	ENST00000244769
chr19	13318672	C	CCTGCTG	290.67	PASS	19	CODON_CHANGE_PLUS _CODON_INSERTION	CACNA1A	ENST00000357018
chr12	121678327	C	CTT	100.24	PASS	15	FRAME_SHIFT	CAMKK2	ENST00000392474
chr11	2906087	G	GGCCGGT	715.46	PASS	15	CODON_CHANGE_PLUS _CODON_INSERTION	CDKN1C	ENST00000313407
chr19	33792731	G	GGCGGGT	241.53	PASS	7	CODON_INSERTION	CEBPA	ENST00000498907
chr4	1388622	T	TCA	784.83	PASS	60	FRAME_SHIFT	CRIPAK	ENST00000324803
chr21	44589920	GC	G	98.55	PASS	21	FRAME_SHIFT	CRYAA	ENST00000398133
chr13	72440658	TGCCGCC	T	527.85	PASS	16	CODON_DELETION	DACH1	ENST00000305425
chr16	67229793	A	ACAGCAGCAG	1265.94	PASS	76	CODON_INSERTION	E2F4	ENST00000379378
chr9	112069507	CT	C	60.34	PASS	6	FRAME_SHIFT	EPB41L4B	ENST00000311609
chr12	8374781	C	CACG	417.95	PASS	22	CODON_INSERTION	FAM90A1	ENST00000307435
chr6	1611802	G	GGGC	177.92	PASS	17	CODON_INSERTION	FOXC1	ENST00000380874
chr20	29625925	C	CTTG	1029.73	PASS	65	CODON_INSERTION	FRG1B	ENST00000278882
chr9	19056367	CT	C	15.87	LowQual; QDFilter	8	FRAME_SHIFT	HAUS6	ENST00000380502
chr14	23744800	ACATCAT	A,ACAT	1399.02	PASS	28	CODON_CHANGE_PLUS _CODON_DELETION	HOMEZ	ENST00000357460
chr1	154842199	G	GGCTGCTGCTGCT	2151.76	PASS	58	CODON_INSERTION	KCNN3	ENST00000539103
chr17	7750177	TACCACC	T,TACCACCACCACC	2524.38	PASS	70	CODON_DELETION	KDM6B	ENST00000254846

Table S1: Novel indels found in genes of MV4-11 resistant cells (continued, Page 2)

CHR	POSITION	REFERENCE BASE	OBSERVED BASE	QUALITY SCORE	FILTER	COV	EFFECT	GENE NAME	TRANSCRIPT ID
chr16	15696480	A	AAGGAAAGAAGGA GGGAGGCAGAG	1835.41	PASS	14	FRAME_SHIFT	KIAA0430	ENST00000344181
chr17	38975343	T	TGC	326.46	PASS	30	FRAME_SHIFT	KRT10	ENST00000269576
chr17	38975344	T	TG	225.18	PASS	32	FRAME_SHIFT	KRT10	ENST00000269576
chr17	39324229	T	TGCAGCAGGTGGT CAG	1847.49	PASS	25	CODON_INSERTION	KRTAP4-3	ENST00000391356
chr15	100252709	CCAGCAG	C	3221.6	PASS	70	CODON_DELETION	MEF2A	ENST00000449277
chr15	90320134	AGGGCAGGGGCAG	A	1971.2	PASS	30	CODON_DELETION	MESP2	ENST00000341735
chr17	1388988	CG	C	50.63	PASS	2	FRAME_SHIFT	MYO1C	ENST00000438665
chr15	23086364	GGCC	G	627.43	PASS	45	CODON_CHANGE_PLUS _CODON_DELETION	NIPA1	ENST00000337435
chr3	98110406	G	GA	43.2	PASS	6	FRAME_SHIFT	OR5K3	ENST00000383695
chr5	141324955	C	CCTGCTGCTG, CCTGCTG	3828.36	PASS	66	CODON_CHANGE_PLUS _CODON_INSERTION	PCDH12	ENST00000231484
chr10	102770293	T	TGCTGCG	2090.38	PASS	111	CODON_INSERTION	PDZD7	ENST00000393462
chr12	113796461	G	GGGCGCTGG CGCT	1082.41	PASS	34	CODON_INSERTION	PLBD2	ENST00000280800
chr12	18854540	ATCCTCCTCCTCC	ATCC,A	1434.75	PASS	33	CODON_CHANGE_PLUS _CODON_DELETION	PLCZ1	ENST00000538330
chr7	131241029	G	GGGCGAC	1060.9	PASS	59	CODON_CHANGE_PLUS _CODON_INSERTION	PODXL	ENST00000544955
chr4	147560457	TGGC	TGGCGGC,T	789.85	PASS	60	CODON_DELETION	POU4F2	ENST00000281321
chrX	16965025	C	CTGA	51.38	PASS	4	CODON_CHANGE_PLUS _CODON_INSERTION	REPS2	ENST00000303843
chrX	16965049	GCTC	G	62.98	PASS	3	CODON_DELETION	REPS2	ENST00000303843
chr11	118757056	GAAA	GA,G	309.13	PASS	20	FRAME_SHIFT	RP11-158I9.5	ENST00000498872
chr15	55477591	GT	G	16.96	LowQual	8	FRAME_SHIFT	RSL24D1	ENST00000260443

Table S1: Novel indels found in genes of MV4-11 resistant cells (continued, Page 3)

CHR	POSITION	REFERENCE BASE	OBSERVED BASE	QUALITY SCORE	FILTER	COV	EFFECT	GENE NAME	TRANSCRIPT ID
chr11	6412853	G	GC	118.72	PASS	24	FRAME_SHIFT	SMPD1	ENST00000299397
chr9	35812098	CACCGCG	C	1697.27	PASS	78	FRAME_SHIFT	SPAG8	ENST00000340291
chr10	135236958	CGCCGCCCCGGCT	C	524.52	PASS	8	CODON_CHANGE_PLUS _CODON_DELETION	SPRN	ENST00000414069
chr6	170871046	ACAGCAGCAG	A	3353.74	PASS	79	CODON_DELETION	TBP	ENST00000540980
chr2	85360828	GGGC	G	64.68	PASS	2	CODON_DELETION	TCF7L1	ENST00000282111
chr11	118406328	CAGTGTGGA	C	2926.05	PASS	33	FRAME_SHIFT	TMEM25	ENST00000533102
chr17	17772622	GAA	G	21.51	LowQual; QDFilter	22	FRAME_SHIFT	TOM1L2	ENST00000537091
chr1	26608889	ACC	A	664.36	PASS	84	FRAME_SHIFT	UBXN11	ENST00000314675
chr3	49349907	C	CA	12.26	LowQual; QDFilter	9	FRAME_SHIFT	USP4	ENST00000416417
chr9	40784154	TCTC	T	214.17	PASS	20	CODON_CHANGE_PLUS _CODON_DELETION	ZNF658	ENST00000441795
chr19	57988666	A	AGCC	1689.18	PASS	109	CODON_CHANGE_PLUS _CODON_INSERTION	ZNF772	ENST00000319969

Table S2: Novel indels found in genes of MOLM-13 resistant cells

CHR	POSITION	REFERENCE BASE	OBSERVED BASE	QUALITY SCORE	FILTER	COV	EFFECT	GENE NAME	TRANSCRIPT ID
chr17	67160234	ATGGTTTTAT	A	645.85	PASS	13	CODON_CHANGE_PLUS _CODON_DELETION	ABCA10	ENST00000269081
chr9	133710722	G	GC	75.27	PASS	3	FRAME_SHIFT	ABL1	ENST00000438426
chr2	69693391	C	CG	1709.82	PASS	169	FRAME_SHIFT	AC114772.1	ENST00000339092
chr5	128797315	T	TCCCGGC	2364.05	PASS	90	CODON_INSERTION	ADAMTS19	ENST00000274487
chr17	18111802	GTTTTTGT	G	2096.39	PASS	96	FRAME_SHIFT	ALKBH5	ENST00000261650
chr2	73613031	T	TGGA	746.96	PASS	37	CODON_INSERTION	ALMS1	ENST00000264448
chr1	213180441	GAA	GAAA,G	121.36	PASS	21	FRAME_SHIFT	ANGEL2	ENST00000310246
chr2	97829003	CAAAAATAA	C	245.62	PASS	12	FRAME_SHIFT	ANKRD36	ENST00000455519
chr11	46699494	GA	G	58.4	QDFilter	35	FRAME_SHIFT	ARHGAP1	ENST00000443332
chr12	7045891	A	ACAGCAG	2213.21	PASS	177	CODON_INSERTION	ATN1	ENST00000356654
chr12	121678327	C	CTTT	117.29	PASS	37	CODON_INSERTION	CAMKK2	ENST00000545538
chr16	838407	GC	G	37.05	PASS	4	FRAME_SHIFT	CHTF18	ENST00000317063
chr11	67209167	T	TG	696.48	PASS	118	FRAME_SHIFT	CORO1B	ENST00000545016
chr14	53619480	T	TGCCGCC	1309.48	PASS	73	CODON_CHANGE_PLUS _CODON_INSERTION	DDHD1	ENST00000323669
chr19	36002402	TGC	T	11.83	LowQual; QDFilter	10	FRAME_SHIFT	DMKN	ENST00000339686
chr16	67229793	A	ACAGCAGCAG	2399.48	PASS	114	CODON_INSERTION	E2F4	ENST00000379378
chr11	19263385	TG	T	11.06	LowQual; QDFilter	9	FRAME_SHIFT	E2F8	ENST00000396159
chr1	21307733	GA	G	84.67	PASS	12	FRAME_SHIFT	EIF4G3	ENST00000400415
chr9	112069507	CT	C	107.01	PASS	10	FRAME_SHIFT	EPB41L4B	ENST00000311609
chr2	186618466	G	GA	157.85	PASS	10	FRAME_SHIFT	FSIP2	ENST00000326147
chr10	46999591	C	CATGAGGGAG	5856.51	PASS	147	CODON_INSERTION	GPRIN2	ENST00000374314
chr17	72839130	A	AGCTCCGGGG	1580.39	PASS	32	CODON_INSERTION	GRIN2C	ENST00000293190
chrX	53652852	T	TGCCGGG	263.94	PASS	6	CODON_INSERTION	HUWE1	ENST00000396323
chr1	154842199	G	GGCTGCTGCTGCTGCT	3289.66	PASS	81	CODON_INSERTION	KCNN3	ENST00000539103

Table S2: Novel indels found in genes of MOLM-13 resistant cells (continued, Page 2)

CHR	POSITION	REFERENCE BASE	OBSERVED BASE	QUALITY SCORE	FILTER	COV	EFFECT	GENE NAME	TRANSCRIPT ID
chr17	7750177	TACCACC	T,TACCACCA CCACC	4863.26	PASS	137	CODON_DELETION	KDM6B	ENST00000254846
chr16	71956511	AATGCCC	A	4673.11	PASS	198	CODON_DELETION	KIAA0174	ENST00000456820
chr3	113376110	TTGC	T	2068.07	PASS	80	CODON_CHANGE_PLUS _CODON_DELETION	KIAA2018	ENST00000316407
chr17	38975265	TTCCGCCGCCGGA	T	1096.46	PASS	32	CODON_DELETION	KRT10	ENST00000269576
chr1	153233991	A	ACTCTGGCGGCGG	2252.27	PASS	59	CODON_INSERTION	LOR	ENST00000368742
chr6	96034869	G	GTATA	448.53	PASS	20	FRAME_SHIFT	MANEA	ENST00000369293
chr15	100252735	AGCAGCAGCC	A	4888.89	PASS	110	CODON_DELETION	MEF2A	ENST00000449277
chr15	90320120	GAGGGGCAGG GGCAAGGGCA GGGGC	G	3626.65	PASS	68	CODON_DELETION	MESP2	ENST00000341735
chr11	12316344	GCTCCTC	G	1141.32	PASS	62	CODON_DELETION	MICALCL	ENST00000256186
chr12	18443817	GTTAATTTCAA	G	456.6	PASS	9	FRAME_SHIFT	PIK3C2G	ENST00000266497
chr6	108076790	T	TG	245.29	PASS	50	FRAME_SHIFT	SCML4	ENST00000369021
chr22	24231164	GA	GAA,G	15.97	LowQual; QDFilter	22	FRAME_SHIFT	SLC2A11	ENST00000398359
chr18	55019963	TCACACA	T,TCACACACACA	915.65	PASS	21	FRAME_SHIFT	ST8SIA3	ENST00000541833
chr1	26608889	ACC	A	1262.52	PASS	121	FRAME_SHIFT	UBXN11	ENST00000314675
chr1	26608892	GGGAC	G	2137.33	PASS	125	FRAME_SHIFT	UBXN11	ENST00000314675
chr16	88599702	CTGGC	C	4833.46	PASS	83	FRAME_SHIFT	ZFPM1	ENST00000319555
chr16	88599700	CTCTGG	C	5831.48	PASS	84	FRAME_SHIFT	ZFPM1	ENST00000319555
chr12	6777069	TTGC	T	2750.85	PASS	113	CODON_CHANGE_PLUS _CODON_DELETION	ZNF384	ENST00000355772
chr19	57956740	C	CA	533.15	PASS	90	FRAME_SHIFT	ZNF749	ENST00000334181
chr19	53454475	C	CACT	1758.59	PASS	89	CODON_CHANGE_PLUS _CODON_INSERTION	ZNF816	ENST00000357666

Table S3: Novel SNPs found in genes of MV4-11 resistant cells

CHR	POSITION	REF BASE	OBS BASE	QUALITY SCORE	FILTER	QD	A,C,G,T	COV	AA CHANGE	EFFECT	GENE NAME	TRANSCRIPT ID
chr22	21403375	C	A	18.28	LowCovFilter; LowQual	3.05	2,4,0,0	6	A184D	NS_CODING	AC002472.13	ENST00000342608
chr7	16129116	A	C	10.43	LowCovFilter; LowQual	10.43	0,1,0,0	1	I113L	NS_CODING	AC006035.2	ENST00000444738
chr19	41416750	T	C	43.97	LowCovFilter	8.79	0,3,0,2	5	E2G	NS_CODING	AC008537.2	ENST00000412510
chr5	795992	C	A	317.37	HaplotypeFilter	2.03	25,130,0,1	155	R177M	NS_CODING	AC026740.1	ENST00000436502
chr5	796328	T	C	243.91	PASS	2.28	0,25,0,82	106	M89V	NS_CODING	AC026740.1	ENST00000436502
chr5	796333	G	A	229.07	PASS	2.57	21,0,68,0	88	P87L	NS_CODING	AC026740.1	ENST00000436502
chr2	48757179	C	G	21.46	LowCovFilter; LowQual; QDFilter	1.26	0,12,5,0	17	A61G	NS_CODING	AC073082.1	ENST00000378305
chr2	48757185	C	G	46.5	LowCovFilter	3.88	0,6,6,0	12	A63G	NS_CODING	AC073082.1	ENST00000378305
chr2	96606944	C	T	26.06	LowCovFilter; LowQual	8.69	0,0,0,3	3	G499R	NS_CODING	AC073995.2	ENST00000456556
chr17	4575893	A	G	19.66	LowQual; QDFilter	0.5	25,0,14,0	39	L651P	NS_CODING	AC091153.1	ENST00000436683
chr7	100550315	T	C	129.52	QDFilter	0.77	0,21,0,148	168	L299P	NS_CODING	AC118759.1	ENST00000379458
chr7	100550342	T	C	25.23	LowQual; QDFilter	0.17	0,16,0,135	150	I308T	NS_CODING	AC118759.1	ENST00000379458
chr16	33961363	C	T	32	QDFilter	0.64	0,42,0,8	50	A360T	NS_CODING	AC136932.2	ENST00000539813
chr16	33961400	G	T	60.39	HaplotypeFilter; QDFilter	1.18	0,1,40,9	50	S347R	NS_CODING	AC136932.2	ENST00000539813
chr2	211085518	T	C	105.33	LowCovFilter	13.17	0,5,0,3	8	H29R	NS_CODING	ACADL	ENST00000233710
chr19	39220197	T	G	13.34	LowQual; QDFilter	0.58	0,0,7,16	23	W527G	NS_CODING	ACTN4	ENST00000445727
chr14	105412009	A	G	69.46	QDFilter	0.62	98,0,14,0	112	M3260T	NS_CODING	AHNAK2	ENST00000333244
chr14	105412066	C	T	206.63	QDFilter	1.95	0,80,0,26	106	R3241H	NS_CODING	AHNAK2	ENST00000333244
chr14	105415431	C	G	23.33	HaplotypeFilter; LowQual; QDFilter	0.14	0,148,19,0	167	M2119I	NS_CODING	AHNAK2	ENST00000333244
chr14	105415433	T	C	38.99	QDFilter	0.24	0,19,0,145	164	M2119V	NS_CODING	AHNAK2	ENST00000333244

Table S3: Novel SNPs found in genes of MV4-11 resistant cells (continued, Page 2)

CHR	POSITION	REF BASE	OBS BASE	QUALITY SCORE	FILTER	QD	A,C,G,T	COV	AA CHANGE	EFFECT	GENE NAME	TRANSCRIPT ID
chr14	105418535	C	G	27.27	LowQual; QDFilter	0.35	0,65,12,0	75	E1085Q	NS_CODING	AHNAK2	ENST00000333244
chr9	33541216	G	C	93.34	QDFilter	1.61	0,7,51,0	58	G294R	NS_CODING	ANKRD18B	ENST00000290943
chr2	97820434	T	G	27.71	LowQual; QDFilter	0.77	0,0,10,26	35	F406V	NS_CODING	ANKRD36	ENST00000420699
chr1	10490285	T	C	80.28	LowCovFilter	20.07	0,3,0,1	4	NA	START_GAINED	APITD1-CORT	ENST00000400900
chr9	33386146	C	A	113.68	PASS	2.03	9,47,0,0	56	V151F	NS_CODING	AQP7	ENST00000379506
chrX	100743632	G	T	132.83	PASS	5.31	0,0,17,8	25	G123V	NS_CODING	ARMCX4	ENST00000423738
chrX	100749023	A	G	33.42	LowCovFilter	2.39	9,0,5,0	14	E1920G	NS_CODING	ARMCX4	ENST00000423738
chrX	100749053	G	A	14.88	LowCovFilter; LowQual; QDFilter	1.24	3,0,9,0	12	G1930E	NS_CODING	ARMCX4	ENST00000423738
chr8	17930772	C	T	107.68	PASS	2.29	0,39,0,8	47	V63I	NS_CODING	ASAH1	ENST00000314146
chr19	50435918	T	C	195.51	HaplotypeFilter	3.99	0,17,1,31	49	S140P	NS_CODING	ATF5	ENST00000423777
chr19	50435934	T	C	26.1	HaplotypeFilter; LowQual; QDFilter	0.48	0,19,0,35	54	L145P	NS_CODING	ATF5	ENST00000423777
chr19	50435939	T	C	138.56	HaplotypeFilter	2.57	0,16,0,38	54	S147P	NS_CODING	ATF5	ENST00000423777
chrX	138897130	A	C	136.5	LowCovFilter	22.75	0,6,0,0	5	C111W	NS_CODING	ATP11C	ENST00000370557
chr22	32841622	C	A	13.32	LowCovFilter; LowQual	2.66	2,3,0,0	5	M12I	NS_CODING	BPIFC	ENST00000432451
chr19	15355273	A	G	27.34	LowQual; QDFilter	0.52	35,0,18,0	52	S784P	NS_CODING	BRD4	ENST00000263377
chr5	878525	T	A	111.23	QDFilter	1.28	13,0,0,74	87	M290L	NS_CODING	BRD9	ENST00000388890
chr14	78234796	C	T	43.53	LowCovFilter	21.77	0,0,0,2	2	H26Y	NS_CODING	C14orf178	ENST00000355883
chr14	60903757	G	A	56.38	LowCovFilter	5.13	3,0,8,0	11	L524F	NS_CODING	C14orf39	ENST00000321731
chr15	75499207	A	C	20.43	LowCovFilter; LowQual; QDFilter	1.14	14,4,0,0	18	H273P	NS_CODING	C15orf39	ENST00000360639

Table S3: Novel SNPs found in genes of MV4-11 resistant cells (continued, Page 3)

CHR	POSITION	REF BASE	OBS BASE	QUALITY SCORE	FILTER	QD	A,C,G,T	COV	AA CHANGE	EFFECT	GENE NAME	TRANSCRIPT ID
chr17	45451894	G	A	96.43	LowCovFilter	8.77	5,0,6,0	11	V216I	NS_CODING	C17orf57	ENST00000517484
chr17	36829701	C	G	24.87	LowQual; QDFilter	1.13	0,14,8,0	22	A350P	NS_CODING	C17orf96	ENST00000325814
chr19	40834329	T	C	349.23	QDFilter	0.87	0,44,0,359	401	K114E	NS_CODING	C19orf47	ENST00000392035
chr4	99027184	T	C	75.28	LowCovFilter	12.55	0,4,0,2	6	I178V	NS_CODING	C4orf37	ENST00000295268
chr4	99049593	A	G	74.23	LowCovFilter	9.28	5,0,3,0	8	Y125H	NS_CODING	C4orf37	ENST00000295268
chr6	42075097	T	G	16.59	HaplotypeFilter; LowQual; QDFilter	0.52	0,0,5,26	31	S185R	NS_CODING	C6orf132	ENST00000341865
chr6	43969839	C	A	103.55	PASS	3.57	7,22,0,0	29	P101T	NS_CODING	C6orf223	ENST00000439969
chr8	99102249	G	A	856.47	PASS	2.92	49,1,243,0	293	R335H	NS_CODING	C8orf47	ENST00000318528
chr5	39341681	C	A	40.16	QDFilter	1	5,35,0,0	40	G102V	NS_CODING	C9	ENST00000263408
chr9	100080855	G	T	300.63	PASS	3.42	0,0,70,18	87	C398F	NS_CODING	C9orf174	ENST00000411667
chr18	20715824	C	A	10.43	LowCovFilter; LowQual	10.43	1,0,0,0	1	P33Q	NS_CODING	CABLES1	ENST00000256925
chr17	20768763	T	C	35.15	HaplotypeFilter; QDFilter	0.35	0,13,0,88	99	K211E	NS_CODING	CCDC144NL	ENST00000327925
chr17	20768788	G	T	50.12	QDFilter	0.49	0,0,85,17	100	H202Q	NS_CODING	CCDC144NL	ENST00000327925
chr13	36828237	T	C	94.38	LowCovFilter	4.97	0,5,0,14	19	K120R	NS_CODING	CCDC169	ENST00000239859
chr2	132288362	T	C	13.99	LowQual;QDFilter	0.25	0,14,0,43	57	M103T	NS_CODING	CCDC74A	ENST00000409856
chr14	20784718	G	T	179.67	LowCovFilter	17.97	0,0,3,7	10	P20T	NS_CODING	CCNB1IP1	ENST00000556563
chr14	99976632	G	C	13.71	HaplotypeFilter; LowQual; QDFilter	0.39	0,6,29,0	35	R419P	NS_CODING	CCNK	ENST00000389879
chr1	208063100	G	A	24.32	HaplotypeFilter; LowQual; QDFilter	0.74	14,0,19,0	33	S28F	NS_CODING	CD34	ENST00000367036
chr17	45234645	G	C	13.61	LowCovFilter; LowQual; QDFilter	0.8	0,4,13,0	16	S133C	NS_CODING	CDC27	ENST00000446365

Table S3: Novel SNPs found in genes of MV4-11 resistant cells (continued, Page 4)

CHR	POSITION	REF BASE	OBS BASE	QUALITY SCORE	FILTER	QD	A,C,G,T	COV	AA CHANGE	EFFECT	GENE NAME	TRANSCRIPT ID
chr16	55862791	T	C	32.04	QDFilter	0.2	0,16,0,141	155	I49V	NS_CODING	CES1	ENST00000361503
chr1	203186947	G	C	11.64	LowQual; QDFilter	0.25	0,5,41,0	46	A340G	NS_CODING	CHIT1	ENST00000255427
chr11	89935586	G	T	308.12	LowCovFilter	34.24	0,0,0,9	9	A141D	NS_CODING	CHORDC1	ENST00000529726
chr9	17394536	C	T	110.01	LowCovFilter	22	0,1,0,4	5	T695I	NS_CODING	CNTLN	ENST00000262360
chr9	17494947	G	A	17.06	LowCovFilter; LowQual	3.41	2,0,3,0	5	V1388M	NS_CODING	CNTLN	ENST00000262360
chr16	76523711	A	C	130.14	PASS	3.42	29,9,0,0	37	N598H	NS_CODING	CNTNAP4	ENST00000478060
chr12	51467812	C	G	253.27	PASS	4.44	0,44,13,0	56	D69H	NS_CODING	CSRNP2	ENST00000228515
chr14	39777676	G	C	43.44	LowCovFilter	14.48	0,2,1,0	3	E280Q	NS_CODING	CTAGE5	ENST00000557038
chr20	57582285	C	T	35.76	LowCovFilter	11.92	0,1,0,2	3	NA	START_GAINED	CTSZ	ENST00000217131
chr11	107286943	C	T	50.05	LowCovFilter	25.03	0,0,0,2	2	G537R	NS_CODING	CWF19L2	ENST00000282251
chr6	32006387	A	T	14.29	LowCovFilter; LowQual	7.15	0,0,0,2	2	H63L	NS_CODING	CYP21A2	ENST00000418967
chr3	98540982	A	G	302.59	LowCovFilter	18.91	7,0,9,0	16	V233A	NS_CODING	DCBLD2	ENST00000404023
chr12	31242358	G	A	18.87	LowQual; QDFilter	0.15	17,0,111,0	128	V243M	NS_CODING	DDX11	ENST00000438391
chr12	31256905	T	C	230.82	LowCovFilter	32.97	0,7,0,0	7	C951R	NS_CODING	DDX11	ENST00000407793
chr9	118163563	C	T	1234.19	PASS	30.85	0,0,0,40	38	A60V	NS_CODING	DEC1	ENST00000374016
chr11	111916647	G	A	780.87	PASS	30.03	26,0,0,0	26	D222N	NS_CODING	DLAT	ENST00000537636
chr19	36002386	C	T	49.24	LowCovFilter	7.03	0,4,0,3	7	S282N	NS_CODING	DMKN	ENST00000339686
chr19	36002389	C	T	46.23	LowCovFilter	5.78	0,5,0,3	8	S281N	NS_CODING	DMKN	ENST00000339686
chr19	36002392	C	T	15.92	LowCovFilter; LowQual	2.27	0,5,0,2	7	G280D	NS_CODING	DMKN	ENST00000339686
chr19	36002395	C	T	15.92	LowCovFilter; LowQual	2.27	0,5,0,2	7	G279D	NS_CODING	DMKN	ENST00000339686
chr19	36002401	C	T	13.02	LowCovFilter; LowQual; QDFilter	1.63	0,6,0,2	8	S277N	NS_CODING	DMKN	ENST00000339686

Table S3: Novel SNPs found in genes of MV4-11 resistant cells (continued, Page 5)

CHR	POSITION	REF BASE	OBS BASE	QUALITY SCORE	FILTER	QD	A,C,G,T	COV	AA CHANGE	EFFECT	GENE NAME	TRANSCRIPT ID
chr1	225373072	C	T	138.19	LowCovFilter	9.87	0,8,0,6	14	T1445M	NS_CODING	DNAH14	ENST00000445597
chr2	183605077	C	A	62.46	LowCovFilter	5.21	5,7,0,0	12	L301I	NS_CODING	DNAJC10	ENST00000392392
chr16	2287496	T	C	72.08	FSFilter; HaplotypeFilter; QDFilter	0.34	0,46,1,162	208	L146P	NS_CODING	DNASE1L2	ENST00000320700
chr7	153750140	G	A	169.41	PASS	4.98	8,0,26,0	34	E79K	NS_CODING	DPP6	ENST00000377770
chr12	63964599	T	C	205.1	PASS	2.36	0,19,1,67	84	I647V	NS_CODING	DPY19L2	ENST00000324472
chr1	245133579	G	C	33.17	HaplotypeFilter; QDFilter	0.66	0,11,39,0	50	R52P	NS_CODING	EFCAB2	ENST00000366522
chr8	21924297	C	T	84.34	LowCovFilter	28.11	0,0,0,3	3	NA	START_GAINED	EPB49	ENST00000517418
chr3	185797720	T	G	121.91	QDFilter	1.94	0,0,25,38	63	H179P	NS_CODING	ETV5	ENST00000306376
chr3	185797727	C	G	30.27	HaplotypeFilter; QDFilter	0.5	0,40,21,0	61	A177P	NS_CODING	ETV5	ENST00000306376
chr20	26061956	C	T	37.83	QDFilter	1.64	2,17,0,4	23	A103V	NS_CODING	FAM182A	ENST00000246000
chr20	26062002	G	C	61.47	LowCovFilter	8.78	0,4,3,0	7	Q118H	NS_CODING	FAM182A	ENST00000246000
chr20	26061877	G	A	217.9	PASS	4.84	13,0,32,0	44	G18R	NS_CODING	FAM182A	ENST00000415411
chr20	26061880	A	G	223.95	PASS	5.09	31,0,13,0	43	I19V	NS_CODING	FAM182A	ENST00000415411
chr20	26061884	C	G	223.95	PASS	5.21	0,27,16,0	42	S20C	NS_CODING	FAM182A	ENST00000415411
chr20	26061859	G	T	172.1	PASS	4	0,0,26,17	39	E12*	STOP_GAINED	FAM182A	ENST00000415411
chr20	26061865	G	T	62.79	QDFilter	1.46	0,0,37,6	42	E14*	STOP_GAINED	FAM182A	ENST00000415411
chr20	25755526	C	T	40.22	LowCovFilter	4.02	0,7,0,3	10	G144R	NS_CODING	FAM182B	ENST00000376403
chr10	51827934	G	A	175.71	LowCovFilter	14.64	7,0,5,0	12	V24M	NS_CODING	FAM21A	ENST00000434114
chr10	47915891	C	A	144.03	LowCovFilter	20.58	7,0,0,0	7	S270Y	NS_CODING	FAM21B	ENST00000535219
chr10	46254776	A	C	24.88	LowCovFilter; LowQual	4.15	3,2,0,1	6	Y433S	NS_CODING	FAM21C	ENST00000436993
chr1	152276660	G	C	86.96	QDFilter	1.02	0,21,64,0	85	Q3568E	NS_CODING	FLG	ENST00000368799
chr1	152276671	C	T	239.08	PASS	2.6	0,66,0,26	92	R3564H	NS_CODING	FLG	ENST00000368799
chr1	152276699	A	G	35.72	QDFilter	0.31	87,0,28,0	115	W3555R	NS_CODING	FLG	ENST00000368799

Table S3: Novel SNPs found in genes of MV4-11 resistant cells (continued, Page 6)

CHR	POSITION	REF BASE	OBS BASE	QUALITY SCORE	FILTER	QD	A,C,G,T	COV	AA CHANGE	EFFECT	GENE NAME	TRANSCRIPT ID
chr1	152280646	C	T	47.49	QDFilter	0.24	0,175,0,25	200	R2239Q	NS_CODING	FLG	ENST00000368799
chr1	152280649	G	C	150.54	QDFilter	0.75	0,26,174,0	200	P2238R	NS_CODING	FLG	ENST00000368799
chr1	152280665	G	A	177.38	QDFilter	0.79	31,0,193,0	224	P2233S	NS_CODING	FLG	ENST00000368799
chr1	152280671	A	C	117.54	QDFilter	0.49	212,30,0,0	242	S2231A	NS_CODING	FLG	ENST00000368799
chr1	152280688	A	G	135.13	QDFilter	0.49	241,0,32,0	273	V2225A	NS_CODING	FLG	ENST00000368799
chr1	152280670	G	T	159.88	QDFilter	0.67	0,0,207,30	237	S2231*	STOP_GAINED	FLG	ENST00000368799
chr1	240370940	C	T	92.46	LowCovFilter	7.11	0,8,0,5	13	P943L	NS_CODING	FMN2	ENST00000319653
chr13	49772578	C	A	621.95	PASS	4.51	29,109,0,0	138	P888H	NS_CODING	FNDC3A	ENST00000337156
chr20	29628263	A	G	23.14	HaplotypeFilter; LowQual; QDFilter	0.06	383,0,26,0	345	I89V	NS_CODING	FRG1B	ENST00000278882
chr20	29625965	G	A	39.6	HaplotypeFilter; QDFilter	0.43	11,0,81,0	87	W70*	STOP_GAINED	FRG1B	ENST00000278882
chr14	44974189	C	A	66.57	LowCovFilter	9.51	3,4,0,0	7	A561S	NS_CODING	FSCB	ENST00000537803
chr2	186620953	T	G	12.29	LowCovFilter; LowQual; QDFilter	0.82	0,0,4,11	14	D342E	NS_CODING	FSIP2	ENST00000326147
chr5	39119723	C	A	144.39	LowCovFilter	16.04	5,4,0,0	9	V672F	NS_CODING	FYB	ENST00000351578
chr11	77937657	C	G	47.26	QDFilter	0.5	0,70,22,1	89	R316P	NS_CODING	GAB2	ENST00000340149
chr3	81698130	T	C	179.69	LowCovFilter	19.97	0,6,0,3	9	R149G	NS_CODING	GBE1	ENST00000489715
chr16	81129822	G	A	141.66	LowCovFilter	17.71	5,0,3,0	7	S21L	NS_CODING	GCSH	ENST00000315467
chrX	30725698	T	C	12.05	LowCovFilter; LowQual	12.05	0,1,0,0	1	C130R	NS_CODING	GK	ENST00000451432
chr19	48197501	T	C	22.51	LowCovFilter; LowQual; QDFilter	1.88	0,3,0,9	12	S805P	NS_CODING	GLTSCR1	ENST00000396720
chr1	6310024	C	T	41.88	LowCovFilter	10.47	0,1,0,3	4	V402M	NS_CODING	GPR153	ENST00000377893
chr4	94411823	G	A	259.74	PASS	9.28	11,0,17,0	28	R536Q	NS_CODING	GRID2	ENST00000510992
chr7	6590670	C	T	32.99	LowCovFilter	8.25	0,1,0,3	3	R133H	NS_CODING	GRID2IP	ENST00000457091

Table S3: Novel SNPs found in genes of MV4-11 resistant cells (continued, Page 7)

CHR	POSITION	REF BASE	OBS BASE	QUALITY SCORE	FILTER	QD	A,C,G,T	COV	AA CHANGE	EFFECT	GENE NAME	TRANSCRIPT ID
chr6	102337567	T	C	534.95	PASS	5.81	0,28,0,64	88	V125A	NS_CODING	GRIK2	ENST00000436862
chr19	1009585	C	G	142.57	LowCovFilter	10.18	0,8,6,0	14	P1039R	NS_CODING	GRIN3B	ENST00000234389
chr5	178412628	C	A	29.69	LowCovFilter; LowQual	2.12	4,10,0,0	14	G893*	STOP_GAINED	GRM6	ENST00000319065
chr14	25101589	G	C	29.45	LowCovFilter; LowQual	3.27	0,3,6,0	8	P128A	NS_CODING	GZMB	ENST00000382542
chr16	778954	T	G	42.6	HaplotypeFilter; QDFilter	0.76	0,0,15,41	56	V220G	NS_CODING	HAGHL	ENST00000341413
chr12	123200527	T	C	44.76	QDFilter	0.79	0,7,0,50	57	H253R	NS_CODING	HCAR3	ENST00000528880
chr2	242169207	A	C	51.24	QDFilter	0.93	39,16,0,0	55	V118G	NS_CODING	HDLBP	ENST00000442730
chr2	242169211	T	C	29.72	LowQual; QDFilter	0.51	0,17,0,41	58	R117G	NS_CODING	HDLBP	ENST00000442730
chr10	93221951	A	G	160.22	LowCovFilter	12.32	5,0,8,0	13	M204V	NS_CODING	HECTD2	ENST00000371681
chr1	91742541	C	T	102.03	LowCovFilter	6.8	0,9,0,6	15	C1157Y	NS_CODING	HFM1	ENST00000370425
chr1	12907533	T	C	202.93	LowCovFilter	11.94	0,8,0,9	17	I204V	NS_CODING	HNRNPCL1	ENST00000317869
chr16	71237702	C	T	215.2	LowCovFilter	11.33	0,11,0,8	19	C8Y	NS_CODING	HYDIN	ENST00000539973
chr5	52229745	T	G	139.15	LowCovFilter	7.73	0,0,6,12	18	I961M	NS_CODING	ITGA1	ENST00000282588
chr3	67058348	G	A	23.09	LowCovFilter; LowQual	3.85	3,0,3,0	6	E423K	NS_CODING	KBTBD8	ENST00000295568
chr8	36767024	T	C	180.92	PASS	5.03	0,9,0,27	36	W768R	NS_CODING	KCNU1	ENST00000399881
chr12	498247	A	C	31.75	HaplotypeFilter; QDFilter	0.32	68,27,0,2	96	V4G	NS_CODING	KDM5A	ENST00000261253
chr17	7751144	G	C	52.15	LowCovFilter	3.73	0,6,8,0	14	R513P	NS_CODING	KDM6B	ENST00000254846
chr19	55286773	A	G	12.1	LowQual; QDFilter	0.14	73,0,13,0	86	K176R	NS_CODING	KIR2DL1	ENST00000291633
chr17	38975163	A	T	11.17	HaplotypeFilter; LowQual; QDFilter	0.24	39,2,0,5	46	Y542N	NS_CODING	KRT10	ENST00000269576
chr17	38975166	C	T	102.09	HaplotypeFilter	2.17	0,39,0,8	47	G541S	NS_CODING	KRT10	ENST00000269576
chr21	45971109	G	A	43.7	QDFilter	0.77	11,0,45,0	56	S78L	NS_CODING	KRTAP10-2	ENST00000391621

Table S3: Novel SNPs found in genes of MV4-11 resistant cells (continued, Page 8)

CHR	POSITION	REF BASE	OBS BASE	QUALITY SCORE	FILTER	QD	A,C,G,T	COV	AA CHANGE	EFFECT	GENE NAME	TRANSCRIPT ID
chr21	45971152	G	A	137.09	PASS	2.11	17,0,48,0	65	P64S	NS_CODING	KRTAP10-2	ENST00000391621
chr17	39197549	G	C	64.04	QDFilter	0.73	0,10,78,0	88	S34C	NS_CODING	KRTAP1-1	ENST00000306271
chr17	39197538	G	T	79.98	QDFilter	0.94	0,0,75,10	85	R28S	NS_CODING	KRTAP1-1	ENST00000543328
chr17	39324333	T	A	117.96	QDFilter	1.57	16,0,0,59	75	Q31L	NS_CODING	KRTAP4-3	ENST00000391356
chr21	40794905	T	C	568.72	PASS	23.7	0,18,0,6	24	I278M	NS_CODING	LCA5L	ENST00000288350
chr2	100938226	G	C	51.75	LowCovFilter	25.88	0,2,0,0	2	D110E	NS_CODING	LONRF2	ENST00000393437
chr19	51022077	G	T	540.11	QDFilter	1.67	0,0,287,37	323	T298K	NS_CODING	LRRC4B	ENST00000389201
chr12	85438499	G	A	29.74	LowCovFilter; LowQual	4.96	2,0,4,0	6	C83Y	NS_CODING	LRRIQ1	ENST00000256007
chrX	26212316	G	T	35.63	LowCovFilter	17.82	0,0,0,2	2	G118V	NS_CODING	MAGEB6	ENST00000379034
chrX	26212324	G	T	35.63	LowCovFilter	17.82	0,0,0,2	2	G121C	NS_CODING	MAGEB6	ENST00000379034
chrX	26212325	G	A	35.63	LowCovFilter	17.82	2,0,0,0	2	G121D	NS_CODING	MAGEB6	ENST00000379034
chrX	140994062	C	G	133.13	QDFilter	1.73	0,66,11,0	77	T291S	NS_CODING	MAGEC1	ENST00000285879
chrX	140994069	T	A	118.1	QDFilter	1.41	11,0,0,73	84	S293R	NS_CODING	MAGEC1	ENST00000285879
chrX	140994074	T	C	124.13	QDFilter	1.48	0,11,0,73	84	F295S	NS_CODING	MAGEC1	ENST00000285879
chrX	140994085	C	G	26.83	LowQual; QDFilter	0.33	0,72,9,0	81	P100A	NS_CODING	MAGEC1	ENST00000370510
chr18	48256037	C	T	472.31	PASS	5.31	0,70,0,19	89	P315L	NS_CODING	MAPK4	ENST00000540640
chr10	45959741	G	A	1736.33	PASS	13.67	65,0,62,0	126	P63L	NS_CODING	MARCH8	ENST00000319836
chr22	40814743	C	G	67.65	PASS	2.71	0,16,9,0	25	A517P	NS_CODING	MKL1	ENST00000402042
chr7	151945334	T	C	24.48	LowQual; QDFilter	0.2	0,28,0,94	120	N729D	NS_CODING	MLL3	ENST00000262189
chr11	102595492	G	A	1089.81	PASS	36.33	30,0,0,0	30	T32I	NS_CODING	MMP8	ENST00000236826
chr1	17086941	C	T	10.54	LowQual; QDFilter	0.34	0,27,0,4	31	R128H	NS_CODING	MST1P9	ENST00000334998
chr1	11168265	T	G	21.68	LowQual; QDFilter	0.72	0,0,4,26	30	E2536A	NS_CODING	MTOR	ENST00000361445
chr7	100634905	A	T	50.82	QDFilter	0.5	89,0,0,13	101	H354L	NS_CODING	MUC12	ENST00000536621

Table S3: Novel SNPs found in genes of MV4-11 resistant cells (continued, Page 9)

CHR	POSITION	REF BASE	OBS BASE	QUALITY SCORE	FILTER	QD	A,C,G,T	COV	AA CHANGE	EFFECT	GENE NAME	TRANSCRIPT ID
chr11	1093549	G	C	64.88	HaplotypeFilter; QDFilter	0.3	5,35,174,0	212	V1746L	NS_CODING	MUC2	ENST00000359061
chr3	195511973	A	G	19.83	LowQual; QDFilter	0.34	50,1,7,0	58	S2160P	NS_CODING	MUC4	ENST00000463781
chr11	1016576	G	C	831.98	FSFilter; HaplotypeFilter	3.89	0,56,142,6	203	H2075Q	NS_CODING	MUC6	ENST00000421673
chr11	1016959	T	C	138.47	FSFilter; HaplotypeFilter; QDFilter	0.18	4,78,0,677	755	R1948G	NS_CODING	MUC6	ENST00000421673
chr11	1017280	G	T	241.79	HaplotypeFilter; QDFilter	0.64	0,0,333,46	379	P1841T	NS_CODING	MUC6	ENST00000421673
chr11	1017294	A	T	194	HaplotypeFilter; QDFilter	0.41	417,0,1,55	472	L1836H	NS_CODING	MUC6	ENST00000421673
chr11	1017892	C	T	85.06	HaplotypeFilter; QDFilter	0.18	1,399,34,46	477	A1637T	NS_CODING	MUC6	ENST00000421673
chr11	1017894	T	C	32.84	FSFilter; HaplotypeFilter; QDFilter	0.07	1,43,0,436	477	H1636R	NS_CODING	MUC6	ENST00000421673
chr11	1017898	T	G	221.28	FSFilter; HaplotypeFilter; QDFilter	0.46	0,0,49,437	483	T1635P	NS_CODING	MUC6	ENST00000421673
chr11	1017988	G	T	62.94	HaplotypeFilter; QDFilter	0.22	0,1,255,29	282	P1605T	NS_CODING	MUC6	ENST00000421673
chr11	1018042	A	G	88.12	HaplotypeFilter; QDFilter	0.34	239,0,24,0	261	S1587P	NS_CODING	MUC6	ENST00000421673
chr11	1018341	G	A	48.6	HaplotypeFilter; QDFilter	0.25	29,0,163,2	192	P1487L	NS_CODING	MUC6	ENST00000421673
chr19	17322755	T	C	45.07	HaplotypeFilter; QDFilter	0.43	0,27,0,78	105	L382P	NS_CODING	MYO9B	ENST00000319396
chr19	17322760	A	C	119.13	FSFilter; HaplotypeFilter; QDFilter	1.17	77,25,0,0	102	T384P	NS_CODING	MYO9B	ENST00000319396
chr19	17322763	G	C	17.84	LowQual; QDFilter	0.18	0,17,80,1	98	V385L	NS_CODING	MYO9B	ENST00000319396

Table S3: Novel SNPs found in genes of MV4-11 resistant cells (continued, Page 10)

CHR	POSITION	REF BASE	OBS BASE	QUALITY SCORE	FILTER	QD	A,C,G,T	COV	AA CHANGE	EFFECT	GENE NAME	TRANSCRIPT ID
chr3	175345143	C	G	118.99	LowCovFilter	17	0,3,4,0	7	P622R	NS_CODING	NAALADL2	ENST00000454872
chr12	57111948	G	T	154.34	LowCovFilter	22.05	0,0,1,6	7	H1122Q	NS_CODING	NACA	ENST00000454682
chr12	57111982	G	A	145.36	LowCovFilter	10.38	7,0,7,0	13	P1111L	NS_CODING	NACA	ENST00000454682
chr16	2011653	A	C	27.31	LowCovFilter; LowQual; QDFilter	1.95	8,6,0,0	14	H142P	NS_CODING	NDUFB10	ENST00000543683
chr3	52797634	G	C	104.64	LowCovFilter	11.63	0,4,5,0	9	P136A	NS_CODING	NEK4	ENST00000461689
chr4	85419237	A	G	41.49	QDFilter	0.48	65,0,20,0	84	S49P	NS_CODING	NKX6-1	ENST00000295886
chr1	120611498	G	C	107.61	QDFilter	0.8	0,36,98,0	133	P191A	NS_CODING	NOTCH2	ENST00000538680
chr1	120611554	T	C	107.76	QDFilter	0.54	0,22,0,176	197	N172S	NS_CODING	NOTCH2	ENST00000538680
chr7	25267934	T	C	132.1	LowCovFilter	33.03	0,4,0,0	4	D42G	NS_CODING	NPVF	ENST00000222674
chr14	79454391	T	G	25.52	LowQual; QDFilter	0.65	0,0,7,32	39	S1046A	NS_CODING	NRXN3	ENST00000332068
chrX	51075746	C	T	27.47	LowQual; QDFilter	0.7	0,34,0,5	37	NA	START_GAINED	NUDT10	ENST00000356450
chr16	4744412	A	G	180.18	FSFilter; HaplotypeFilter	2.02	66,1,22,0	89	E196G	NS_CODING	NUDT16L1	ENST00000405142
chr16	4744417	C	G	206.77	FSFilter; HaplotypeFilter	2.35	0,69,19,0	88	R198G	NS_CODING	NUDT16L1	ENST00000405142
chr19	464136	C	G	10.12	LowQual; QDFilter	0.24	1,28,13,0	41	R157P	NS_CODING	ODF3L2	ENST00000382696
chr11	123893988	C	T	34.69	LowCovFilter	4.96	0,5,0,2	7	A90V	NS_CODING	OR10G9	ENST00000375024
chr1	248224489	A	G	70.88	QDFilter	0.37	165,0,28,0	189	Q169R	NS_CODING	OR2L3	ENST00000359959
chr11	56143898	G	T	14.27	LowQual; QDFilter	0.25	0,0,52,6	58	A267S	NS_CODING	OR8U1	ENST00000302270
chr11	56143907	A	G	35.18	QDFilter	0.68	46,0,6,0	52	T270A	NS_CODING	OR8U1	ENST00000302270
chr14	20919551	C	T	40.94	LowCovFilter	13.65	0,1,0,2	3	G158R	NS_CODING	OSGEP	ENST00000488532
chr4	4190595	G	C	14.47	LowQual; QDFilter	0.12	0,19,99,0	118	P592A	NS_CODING	OTOP1	ENST00000296358
chr8	101719004	G	A	28.87	LowQual;QDFilter	0.29	14,0,84,0	94	R2C	NS_CODING	PABPC1	ENST00000517990

Table S3: Novel SNPs found in genes of MV4-11 resistant cells (continued, Page 11)

CHR	POSITION	REF BASE	OBS BASE	QUALITY SCORE	FILTER	QD	A,C,G,T	COV	AA CHANGE	EFFECT	GENE NAME	TRANSCRIPT ID
chr8	101721429	T	G	16.88	LowQual; QDFilter	0.33	0,0,6,43	49	Y378S	NS_CODING	PABPC1	ENST00000519004
chr8	101721430	A	G	11.13	LowQual; QDFilter	0.22	45,0,6,0	51	Y378H	NS_CODING	PABPC1	ENST00000519004
chr8	101721442	G	A	26.46	LowQual; QDFilter	0.52	7,0,44,0	51	R374C	NS_CODING	PABPC1	ENST00000519004
chr8	101721451	T	A	14.56	LowQual; QDFilter	0.27	7,0,0,47	54	T371S	NS_CODING	PABPC1	ENST00000519004
chr3	196529902	G	C	25.99	LowQual; QDFilter	0.43	0,8,53,0	60	Q101H	NS_CODING	PAK2	ENST00000327134
chr5	102338777	G	A	24.96	LowQual; QDFilter	0.78	5,0,27,0	32	G445R	NS_CODING	PAM	ENST00000348126
chr22	50616639	G	C	13.03	LowCovFilter; LowQual; QDFilter	1.09	0,4,8,0	11	A500P	NS_CODING	PANX2	ENST00000159647
chr16	50257107	G	C	226.38	LowCovFilter	22.64	0,7,3,0	10	C303S	NS_CODING	PAPD5	ENST00000357464
chr13	25000617	C	G	122.04	LowCovFilter	20.34	0,1,5,0	6	A1656P	NS_CODING	PARP4	ENST00000381989
chr10	55755471	C	A	544.08	PASS	15.11	20,16,0,0	36	A547S	NS_CODING	PCDH15	ENST00000409834
chr5	140558212	A	C	75.88	LowCovFilter	18.97	1,3,0,0	4	K199N	NS_CODING	PCDHB8	ENST00000239444
chr1	55524237	G	A	210.56	LowCovFilter	26.32	8,0,0,0	8	V474I	NS_CODING	PCSK9	ENST00000302118
chr1	186418600	C	T	110.46	LowCovFilter	8.5	0,7,0,6	13	NA	START_GAINED	PDC	ENST00000391997
chr19	43983736	T	G	102.05	PASS	2.76	0,0,17,20	37	T499P	NS_CODING	PHLDB3	ENST00000292140
chr11	64032540	A	C	22.87	HaplotypeFilter; LowQual; QDFilter	0.51	35,10,0,0	45	T857P	NS_CODING	PLCB3	ENST00000325234
chr12	19441028	A	G	17.95	LowCovFilter; LowQual	2.99	4,0,2,0	6	K318R	NS_CODING	PLEKHA5	ENST00000412219
chr19	4511723	C	G	138.62	QDFilter	0.74	0,169,18,0	187	G736A	NS_CODING	PLIN4	ENST00000301286
chr3	145803142	T	A	84.43	LowCovFilter	28.14	3,0,0,0	3	K9I	NS_CODING	PLOD2	ENST00000461497
chr1	205817018	T	C	10.43	LowCovFilter; LowQual	10.43	0,1,0,0	1	H84R	NS_CODING	PM20D1	ENST00000367136

Table S3: Novel SNPs found in genes of MV4-11 resistant cells (continued, Page 12)

CHR	POSITION	REF BASE	OBS BASE	QUALITY SCORE	FILTER	QD	A,C,G,T	COV	AA CHANGE	EFFECT	GENE NAME	TRANSCRIPT ID
chr7	94740697	G	T	367.68	PASS	9.94	0,0,19,18	35	E508*	STOP_GAINED	PPP1R9A	ENST00000289495
chr12	11035274	G	T	147.88	PASS	2.28	0,0,53,12	65	L42I	NS_CODING	PRH1	ENST00000428168
chr21	48068485	C	A	1135.49	PASS	13.85	41,41,0,0	82	L34M	NS_CODING	PRMT2	ENST00000379844
chr17	1585191	C	A	256.76	PASS	2.02	19,108,0,0	124	Q192H	NS_CODING	PRPF8	ENST00000304992
chr2	240982171	A	C	16.26	LowQual; QDFilter	0.74	19,3,0,0	22	S77A	NS_CODING	PRR21	ENST00000408934
chr2	240982219	A	G	110.33	PASS	5.02	16,0,6,0	22	S61P	NS_CODING	PRR21	ENST00000408934
chr7	142458929	G	C	18.75	LowQual; QDFilter	0.6	0,5,26,0	31	K70N	NS_CODING	PRSS1	ENST00000486171
chr7	142458938	T	A	30.4	QDFilter	1.09	5,0,1,22	28	F73L	NS_CODING	PRSS1	ENST00000486171
chr7	56087399	G	A	43.29	QDFilter	1.2	7,0,29,0	36	P57S	NS_CODING	PSPH	ENST00000275605
chr7	56087423	G	A	64.98	QDFilter	1.97	8,0,25,0	33	R49W	NS_CODING	PSPH	ENST00000275605
chr1	44063427	G	C	12.82	HaplotypeFilter; LowQual; QDFilter	0.27	0,7,41,0	48	A608P	NS_CODING	PTPRF	ENST00000359947
chr12	80933609	A	G	71.6	LowCovFilter	10.23	4,0,3,0	7	I1010V	NS_CODING	PTPRQ	ENST00000266688
chr3	141259432	T	A	14.06	LowCovFilter; LowQual; QDFilter	1	2,0,0,12	14	C170S	NS_CODING	RASA2	ENST00000286364
chr17	77111772	T	G	182.68	HaplotypeFilter	6.77	0,0,11,15	26	Q9P	NS_CODING	RBFOX3	ENST00000338834
chrX	135956575	G	A	38.31	LowCovFilter; QDFilter	1.92	8,0,12,0	20	P288L	NS_CODING	RBMX	ENST00000449161
chrX	135958704	G	C	41.55	LowCovFilter	2.6	0,7,9,0	12	P154A	NS_CODING	RBMX	ENST00000449161
chrX	135958730	C	A	61.84	LowCovFilter	2.81	9,13,0,0	18	G145V	NS_CODING	RBMX	ENST00000449161
chr14	68193731	G	A	218.25	LowCovFilter	18.19	8,0,4,0	12	R161Q	NS_CODING	RDH12	ENST00000267502
chrX	16965001	C	T	24.85	LowCovFilter; LowQual	6.21	0,2,0,2	4	A6V	NS_CODING	REPS2	ENST00000303843
chrX	16965031	C	A	24.85	LowCovFilter; LowQual	6.21	2,2,0,0	4	A16E	NS_CODING	REPS2	ENST00000303843
chr2	102068872	T	A	169.37	LowCovFilter	12.1	7,0,0,7	14	R79W	NS_CODING	RFX8	ENST00000376826

Table S3: Novel SNPs found in genes of MV4-11 resistant cells (continued, Page 13)

CHR	POSITION	REF BASE	OBS BASE	QUALITY SCORE	FILTER	QD	A,C,G,T	COV	AA CHANGE	EFFECT	GENE NAME	TRANSCRIPT ID
chr8	54870974	A	C	262	PASS	2.79	70,24,0,0	94	K109Q	NS_CODING	RGS20	ENST00000522225
chr5	158630629	G	T	20.51	LowQual; QDFilter	0.64	0,0,27,5	32	N13K	NS_CODING	RNF145	ENST00000520638
chr5	175957905	T	G	26.33	HaplotypeFilter; LowQual; QDFilter	0.59	0,0,13,32	45	T114P	NS_CODING	RNF44	ENST00000537487
chr5	175957910	T	G	134.62	PASS	3.28	0,0,16,25	41	Q112P	NS_CODING	RNF44	ENST00000537487
chr17	15517237	G	C	56.77	QDFilter	1.01	0,12,44,0	56	L261V	NS_CODING	RP11-385D13.1	ENST00000261644
chr17	15517284	G	C	39.08	QDFilter	0.51	0,13,64,0	77	A245G	NS_CODING	RP11-385D13.1	ENST00000261644
chr1	1355796	C	T	85.08	LowCovFilter	28.36	0,0,0,3	3	R129H	NS_CODING	RP4-758J18.6	ENST00000537107
chr20	17639804	T	C	150.46	HaplotypeFilter; QDFilter	0.51	0,37,0,260	295	K450R	NS_CODING	RRBP1	ENST00000246043
chr14	101347299	T	G	138.74	HaplotypeFilter	3.65	0,0,19,18	37	H1276P	NS_CODING	RTL1	ENST00000534062
chr15	77176200	A	T	33.59	LowCovFilter	6.72	3,0,0,2	5	V41D	NS_CODING	SCAPER	ENST00000303521
chr14	31099738	A	G	98.63	LowCovFilter	7.59	9,0,4,0	13	K38R	NS_CODING	SCFD1	ENST00000557076
chr12	109017650	G	C	80.56	QDFilter	0.59	0,16,120,0	136	P135R	NS_CODING	SELPLG	ENST00000388962
chr17	75398498	C	T	3326.98	PASS	15.92	0,92,0,117	207	P127L	NS_CODING	SEPT9	ENST00000329047
chr17	75494705	A	G	1645.84	PASS	29.92	0,0,55,0	55	M325V	NS_CODING	SEPT9	ENST00000431235
chr17	75495397	T	C	3622.92	PASS	35.87	0,101,0,0	101	W134R	NS_CODING	SEPT9	ENST00000543686
chr18	61575232	A	G	40.25	LowCovFilter	6.71	3,0,3,0	6	NA	START_GAINED	SERPINB10	ENST00000238508
chr17	27283235	C	G	107.74	PASS	4.68	0,11,12,0	23	R82P	NS_CODING	SEZ6	ENST00000540632
chr17	27283241	C	G	20.49	LowQual; QDFilter	0.85	1,14,9,0	24	R80P	NS_CODING	SEZ6	ENST00000540632
chr22	38051454	A	C	94.29	HaplotypeFilter	3.63	15,6,0,5	25	L623F	NS_CODING	SH3BP1	ENST00000357436
chr22	38051461	A	C	210.35	HaplotypeFilter	8.09	7,18,0,0	24	T626P	NS_CODING	SH3BP1	ENST00000357436
chr4	2820055	C	A	14.92	LowCovFilter; LowQual	7.46	2,0,0,0	2	C35*	STOP_GAINED	SH3BP2	ENST00000503393

Table S3: Novel SNPs found in genes of MV4-11 resistant cells (continued, Page 14)

CHR	POSITION	REF BASE	OBS BASE	QUALITY SCORE	FILTER	QD	A,C,G,T	COV	AA CHANGE	EFFECT	GENE NAME	TRANSCRIPT ID
chr3	164714532	C	T	78.81	LowCovFilter	26.27	0,0,0,3	3	M1523I	NS_CODING	SI	ENST00000264382
chr19	51920196	G	T	47.34	QDFilter	0.25	0,0,166,22	188	Q111K	NS_CODING	SIGLEC10	ENST00000530476
chr18	11609811	G	C	107.33	PASS	2.5	0,8,35,0	43	V34L	NS_CODING	SLC35G4	ENST00000424015
chr2	44531284	T	G	37.88	QDFilter	0.62	0,0,13,48	60	F102C	NS_CODING	SLC3A1	ENST00000409380
chr11	6412870	G	C	83.06	HaplotypeFilter	3.78	0,6,15,1	22	S191T	NS_CODING	SMPD1	ENST00000527275
chr2	231248261	C	T	72.9	LowCovFilter	14.58	0,2,0,3	5	T225M	NS_CODING	SP140L	ENST00000243810
chr1	118642236	C	A	51.39	PASS	2.45	5,16,0,0	21	Q274H	NS_CODING	SPAG17	ENST00000336338
chr1	16262465	A	C	15.49	LowCovFilter; LowQual; QDFilter	0.77	15,5,0,0	20	T3244P	NS_CODING	SPEN	ENST00000375759
chr11	47376884	C	T	599.68	PASS	2.19	0,237,0,37	274	G236D	NS_CODING	SPI1	ENST00000378538
chr10	135237196	C	T	108.14	LowCovFilter	21.63	0,1,0,4	5	NA	START_GAINED	SPRN	ENST00000414069
chr19	56029556	C	G	15.63	HaplotypeFilter; LowQual; QDFilter	0.09	0,166,14,1	179	P1305A	NS_CODING	SSC5D	ENST00000389623
chr18	55019921	A	C	88.73	LowCovFilter	17.75	1,4,0,0	5	H56P	NS_CODING	ST8SIA3	ENST00000541833
chr18	55019927	G	C	101.24	LowCovFilter	20.25	0,4,1,0	5	R58P	NS_CODING	ST8SIA3	ENST00000541833
chr2	169103770	C	A	14.92	LowCovFilter; LowQual	7.46	2,0,0,0	2	C59F	NS_CODING	STK39	ENST00000355999
chr10	29784072	G	C	46.23	LowCovFilter	3.85	0,6,6,0	12	P1235A	NS_CODING	SVIL	ENST00000355867
chr14	64640665	C	G	44.32	LowCovFilter	11.08	0,1,3,0	4	L5590V	NS_CODING	SYNE2	ENST00000554584
chr7	105733037	T	C	35.2	LowCovFilter	11.73	0,3,0,0	3	K224E	NS_CODING	SYPL1	ENST00000470347
chr4	104512761	G	T	163.77	LowCovFilter	11.7	0,0,8,6	14	A323E	NS_CODING	TACR3	ENST00000304883
chr12	11174416	A	G	29.78	LowQual; QDFilter	0.52	49,0,8,0	57	L252P	NS_CODING	TAS2R19	ENST00000390673
chr12	11183066	A	T	82.72	QDFilter	0.6	122,0,0,17	138	F290Y	NS_CODING	TAS2R31	ENST00000390675
chr12	11244470	T	C	98.6	QDFilter	1.7	0,11,0,47	57	H120R	NS_CODING	TAS2R43	ENST00000531678
chr15	78369972	G	C	11.55	LowCovFilter; LowQual	11.55	0,1,0,0	1	A8G	NS_CODING	TBC1D2B	ENST00000300584

Table S3: Novel SNPs found in genes of MV4-11 resistant cells (continued, Page 15)

CHR	POSITION	REF BASE	OBS BASE	QUALITY SCORE	FILTER	QD	A,C,G,T	COV	AA CHANGE	EFFECT	GENE NAME	TRANSCRIPT ID
chr6	167592601	G	A	49.3	QDFilter	0.59	9,0,73,0	80	G254R	NS_CODING	TCP10L2	ENST00000366832
chr6	167592605	T	G	48.42	QDFilter	0.59	0,0,9,73	80	V255G	NS_CODING	TCP10L2	ENST00000366832
chr6	167594216	C	T	108.96	LowCovFilter	12.11	0,3,0,6	9	R289W	NS_CODING	TCP10L2	ENST00000366832
chr1	168165788	G	A	155.93	LowCovFilter	12.99	8,0,3,1	12	V174I	NS_CODING	TIPRL	ENST00000367833
chr17	7287538	G	A	92.07	LowCovFilter	10.23	4,0,5,0	9	V278I	NS_CODING	TNK1	ENST00000311668
chr16	1291178	T	C	92.45	PASS	4.2	0,7,0,15	22	V29A	NS_CODING	TPSAB1	ENST00000338844
chr16	1291318	G	C	81.49	PASS	2.33	0,8,27,0	35	V76L	NS_CODING	TPSAB1	ENST00000338844
chr16	1291545	C	T	23.88	LowCovFilter; LowQual	4.78	0,0,0,5	5	T115I	NS_CODING	TPSAB1	ENST00000338844
chr16	1291554	T	C	16.24	LowCovFilter; LowQual	2.71	0,5,0,1	6	I118T	NS_CODING	TPSAB1	ENST00000338844
chr16	1307060	A	G	69.88	QDFilter	0.88	67,0,12,0	79	N166D	NS_CODING	TPSD1	ENST00000397534
chr16	31226445	T	C	11.55	LowCovFilter; LowQual	5.78	0,1,0,1	2	L129P	NS_CODING	TRIM72	ENST00000322122
chr7	72436652	A	G	68.27	LowCovFilter	7.59	0,0,9,0	9	W13R	NS_CODING	TRIM74	ENST00000285805
chr11	101353888	C	A	63.77	QDFilter	1.16	7,48,0,0	55	K318N	NS_CODING	TRPC6	ENST00000348423
chr8	116631738	G	A	48.67	QDFilter	0.22	19,0,201,0	219	A137V	NS_CODING	TRPS1	ENST00000519076
chr18	72997831	A	C	93.51	HaplotypeFilter	2.83	18,14,1,0	33	T112P	NS_CODING	TSHZ1	ENST00000322038
chr18	72997846	A	C	95.56	PASS	2.9	18,14,0,0	32	T117P	NS_CODING	TSHZ1	ENST00000322038
chr18	72997850	G	C	35.44	QDFilter	1.22	0,7,19,3	29	C118S	NS_CODING	TSHZ1	ENST00000322038
chr12	71523134	A	C	125.01	LowCovFilter	13.89	2,7,0,0	9	S130A	NS_CODING	TSPAN8	ENST00000552128
chr21	38525356	T	C	308.97	LowCovFilter	25.75	0,10,0,2	12	M530T	NS_CODING	TTC3	ENST00000540756
chr2	179549988	G	A	123.76	LowCovFilter	30.94	4,0,0,0	4	P9577L	NS_CODING	TTN	ENST00000342992
chr2	179603916	C	A	754.39	PASS	9.67	30,48,0,0	78	D4319Y	NS_CODING	TTN	ENST00000356127
chr1	26608807	T	C	22.56	HaplotypeFilter; LowQual; QDFilter	0.71	0,4,0,28	30	S273G	NS_CODING	UBXN11	ENST00000374223
chr1	26608866	C	G	143.96	QDFilter	1.92	0,64,11,0	74	G253A	NS_CODING	UBXN11	ENST00000374223

Table S3: Novel SNPs found in genes of MV4-11 resistant cells (continued, Page 16)

CHR	POSITION	REF BASE	OBS BASE	QUALITY SCORE	FILTER	QD	A,C,G,T	COV	AA CHANGE	EFFECT	GENE NAME	TRANSCRIPT ID
chr1	26608867	C	A	84.49	QDFilter	1.14	10,64,0,0	73	G253C	NS_CODING	UBXN11	ENST00000374223
chr17	5036274	C	A	38.65	QDFilter	0.35	11,99,1,0	110	H89N	NS_CODING	USP6	ENST00000250066
chr17	5036281	G	C	110.94	QDFilter	1	0,13,98,0	109	S91T	NS_CODING	USP6	ENST00000250066
chrX	8138187	G	T	24.89	LowCovFilter; LowQual	2.77	0,0,7,2	8	D102E	NS_CODING	VCX2	ENST00000317103
chr6	133014444	A	C	33.05	QDFilter	0.79	34,8,0,0	42	F182C	NS_CODING	VNN1	ENST00000367928
chr7	29923888	A	C	40.57	QDFilter	1.5	19,8,0,0	26	I260L	NS_CODING	WIPF3	ENST00000242140
chr17	7197794	C	A	38.87	LowCovFilter	12.96	2,1,0,0	3	G9V	NS_CODING	YBX2	ENST00000007699
chr3	167023738	G	C	111.91	LowCovFilter	13.99	0,4,4,0	8	A444G	NS_CODING	ZBBX	ENST00000392764
chr3	167068256	T	A	64.84	LowCovFilter	5.89	4,0,0,7	11	K131N	NS_CODING	ZBBX	ENST00000392764
chr3	42700902	A	G	43.15	QDFilter	1.11	25,0,14,0	39	E352G	NS_CODING	ZBTB47	ENST00000232974
chr14	24000917	A	G	532.37	PASS	3.83	111,0,28,0	137	M858T	NS_CODING	ZFHX2	ENST00000419474
chr16	72829200	C	A	306.24	PASS	2.78	18,90,2,0	109	E1547*	STOP_GAINED	ZFHX3	ENST00000397992
chr19	58370657	C	A	16.62	LowQual; QDFilter	0.11	41,108,0,0	147	Q250K	NS_CODING	ZNF587	ENST00000376209
chr17	5085179	A	G	40.1	QDFilter	0.35	103,0,13,0	113	V3A	NS_CODING	ZNF594	ENST00000381752
chr4	59350	A	G	25.08	LowQual; QDFilter	0.05	489,0,38,1	519	I11V	NS_CODING	ZNF595	ENST00000509152
chr3	40570842	C	A	160.97	PASS	2.78	10,48,0,0	57	Y19*	STOP_GAINED	ZNF621	ENST00000310898
chrX	22292037	G	A	175.96	PASS	2.07	13,0,72,0	83	R310H	NS_CODING	ZNF645	ENST00000323684
chr19	22363897	T	A	11.14	LowQual; QDFilter	0.21	6,0,0,48	54	K208*	STOP_GAINED	ZNF676	ENST00000397121
chr3	75786598	G	A	16.6	LowCovFilter; LowQual; QDFilter	1.19	3,0,11,0	14	R676W	NS_CODING	ZNF717	ENST00000478296
chr3	75786662	A	T	43.92	LowCovFilter	2.58	13,0,0,4	17	N654K	NS_CODING	ZNF717	ENST00000478296
chr3	75786672	C	T	16.58	LowCovFilter; LowQual; QDFilter	1.11	0,12,0,3	15	S651N	NS_CODING	ZNF717	ENST00000478296

Table S3: Novel SNPs found in genes of MV4-11 resistant cells (continued, Page 17)

CHR	POSITION	REF BASE	OBS BASE	QUALITY SCORE	FILTER	QD	A,C,G,T	COV	AA CHANGE	EFFECT	GENE NAME	TRANSCRIPT ID
chr3	75786681	G	A	52.93	LowCovFilter	3.53	4,0,11,0	15	P648L	NS_CODING	ZNF717	ENST00000478296
chr3	75786684	G	T	22.52	LowCovFilter; LowQual; QDFilter	1.61	0,0,11,3	14	T647K	NS_CODING	ZNF717	ENST00000478296
chr3	75787728	C	T	29.86	LowCovFilter; LowQual	14.93	0,0,0,2	2	R299H	NS_CODING	ZNF717	ENST00000478296
chr3	75790953	C	T	25.99	LowCovFilter; LowQual	8.66	0,1,0,2	3	NA	START_GAINED	ZNF717	ENST00000400845
chr7	76069811	T	C	317.08	PASS	3.11	0,24,0,78	101	S139P	NS_CODING	ZP3	ENST00000416245
chr1	78044477	T	A	10.43	LowCovFilter; LowQual	10.43	1,0,0,0	1	L226F	NS_CODING	ZZZ3	ENST00000370798

Table S4: Novel SNPs found in genes of MOLM-13 resistant cells

CHR	POSITION	REF BASE	OBS BASE	QUALITY SCORE	FILTER	QD	A,C,G,T	COV	AA CHANGE	EFFECT	GENE NAME	TRANSCRIPT ID
chr22	21403375	C	A	19.02	LowCovFilter; LowQual; QDFilter	0.95	6,14,0,0	18	A184D	NS_CODING	AC002472.13	ENST00000342608
chr2	96525640	G	T	11.91	LowCovFilter; LowQual; QDFilter	1.19	0,0,8,2	9	L1289I	NS_CODING	AC073995.2	ENST00000456556
chr2	96614313	C	G	82.96	QDFilter	1.84	0,34,11,0	45	A443P	NS_CODING	AC073995.2	ENST00000456556
chr2	96614322	A	C	98.72	PASS	2.35	32,10,0,0	41	F440V	NS_CODING	AC073995.2	ENST00000456556
chr2	96614325	G	T	121.42	PASS	2.82	0,0,32,11	42	R439S	NS_CODING	AC073995.2	ENST00000456556
chr2	96614339	A	C	65.28	QDFilter	1.52	36,7,0,0	42	L434R	NS_CODING	AC073995.2	ENST00000456556
chr17	4575893	A	G	121.47	FSFilter; HaplotypeFilter	2.58	30,0,16,0	45	L651P	NS_CODING	AC091153.1	ENST00000436683
chr7	100550326	A	G	160.13	QDFilter	0.66	211,0,30,0	240	T303A	NS_CODING	AC118759.1	ENST00000379458
chr7	100550327	C	T	172.07	QDFilter	0.73	0,206,1,30	236	T303I	NS_CODING	AC118759.1	ENST00000379458
chr7	100550338	G	C	188.91	QDFilter	0.78	1,29,212,0	241	G307R	NS_CODING	AC118759.1	ENST00000379458
chr7	100550339	G	A	190.74	QDFilter	0.79	29,0,212,0	240	G307D	NS_CODING	AC118759.1	ENST00000379458
chr7	100550342	T	C	234.8	QDFilter	0.97	0,31,0,210	240	I308T	NS_CODING	AC118759.1	ENST00000379458
chr7	100550348	A	C	178.47	QDFilter	0.72	217,30,0,0	246	N310T	NS_CODING	AC118759.1	ENST00000379458
chr7	100550360	T	C	92.06	QDFilter	0.38	0,26,1,214	239	L314P	NS_CODING	AC118759.1	ENST00000379458
chr7	100550396	G	C	40.48	HaplotypeFilter; QDFilter	0.17	0,24,211,0	234	S326T	NS_CODING	AC118759.1	ENST00000379458
chr17	78978126	G	C	21.13	LowCovFilter; LowQual	7.04	0,2,1,0	3	R23P	NS_CODING	AC127496.1	ENST00000321930
chr16	33961363	C	T	85.99	QDFilter	1.12	0,65,0,12	77	A360T	NS_CODING	AC136932.2	ENST00000539813
chr16	33961400	G	T	79.11	QDFilter	1.2	0,1,54,11	66	S347R	NS_CODING	AC136932.2	ENST00000539813
chr16	33961499	C	T	39.88	QDFilter	0.32	0,111,0,15	125	M314I	NS_CODING	AC136932.2	ENST00000539813
chr16	33961500	A	G	70.33	QDFilter	0.54	114,0,16,0	129	M314T	NS_CODING	AC136932.2	ENST00000539813
chr16	33961539	A	C	84.63	HaplotypeFilter; QDFilter	0.51	145,20,0,0	163	L301R	NS_CODING	AC136932.2	ENST00000539813

Table S4: Novel SNPs found in genes of MOLM-13 resistant cells (continued, Page 2)

CHR	POSITION	REF BASE	OBS BASE	QUALITY SCORE	FILTER	QD	A,C,G,T	COV	AA CHANGE	EFFECT	GENE NAME	TRANSCRIPT ID
chr16	33961855	T	G	30.09	QDFilter	0.7	0,0,8,35	42	T196P	NS_CODING	AC136932.2	ENST00000539813
chr19	36211499	T	C	58.27	QDFilter	1.21	0,15,0,33	48	L417P	NS_CODING	AD000671.1	ENST00000222270
chr19	36212143	T	C	44.91	HaplotypeFilter; QDFilter	0.52	0,18,0,68	86	S632P	NS_CODING	AD000671.1	ENST00000222270
chr4	175899075	C	T	310.52	QDFilter	1.37	0,187,0,39	223	T800I	NS_CODING	ADAM29	ENST00000359240
chr4	175899088	G	T	58.25	QDFilter	0.27	0,2,187,26	213	R804S	NS_CODING	ADAM29	ENST00000359240
chr1	27877785	C	T	160.76	QDFilter	0.89	0,161,0,20	181	R281H	NS_CODING	AHDC1	ENST00000247087
chr14	105418535	C	G	79.71	QDFilter	0.65	0,104,19,0	123	E1085Q	NS_CODING	AHNAK2	ENST00000333244
chr9	117138522	A	C	19.26	HaplotypeFilter; LowQual; QDFilter	0.69	18,9,0,0	27	V522G	NS_CODING	AKNA	ENST00000394574
chr1	16862472	G	C	118.13	QDFilter	0.18	0,55,607,0	756	D65H	NS_CODING	AL355149.1	ENST00000357301
chr9	3453624	T	C	10.43	LowCovFilter; LowQual	10.43	0,1,0,0	1	N38D	NS_CODING	AL365202.1	ENST00000366116
chr1	151682991	T	G	25.3	HaplotypeFilter; LowQual; QDFilter	1.2	0,0,3,18	21	V8G	NS_CODING	AL589765.1	ENST00000442233
chr2	97818264	G	T	51.1	LowCovFilter	2.84	0,0,13,5	18	V396F	NS_CODING	ANKRD36	ENST00000420699
chr2	97828982	C	T	19.21	LowQual; QDFilter	0.84	0,15,0,8	23	A276V	NS_CODING	ANKRD36	ENST00000455519
chr2	97833466	C	T	229.23	PASS	7.39	0,15,0,16	31	A532V	NS_CODING	ANKRD36	ENST00000420699
chr22	16157883	T	C	84.8	PASS	2.83	0,8,0,22	30	K19R	NS_CODING	AP000525.1	ENST00000383146
chr22	16157913	T	G	55.93	PASS	2.54	0,0,6,16	22	K9T	NS_CODING	AP000525.1	ENST00000383146
chr1	10490285	T	C	29.28	LowCovFilter; LowQual	4.18	0,2,0,5	7	NA	START_GAINED	APITD1-CORT	ENST00000400900
chr9	33386510	C	T	54.48	QDFilter	0.68	0,69,0,11	79	A100T	NS_CODING	AQP7	ENST00000297988
chrX	100749023	A	G	28.51	LowCovFilter; LowQual; QDFilter	1.9	9,0,6,0	15	E1920G	NS_CODING	ARMCX4	ENST00000423738
chrX	100749037	G	A	44.2	LowCovFilter	3.68	3,0,9,0	12	A1925T	NS_CODING	ARMCX4	ENST00000423738

Table S4: Novel SNPs found in genes of MOLM-13 resistant cells (continued, Page 3)

CHR	POSITION	REF BASE	OBS BASE	QUALITY SCORE	FILTER	QD	A,C,G,T	COV	AA CHANGE	EFFECT	GENE NAME	TRANSCRIPT ID
chrX	100749077	A	G	65.6	LowCovFilter	3.45	12,0,7,0	19	E1938G	NS_CODING	ARMCX4	ENST00000423738
chr22	30217992	C	T	248.02	LowCovFilter	27.56	0,0,0,9	9	R205K	NS_CODING	ASCC2	ENST00000412689
chr8	62550895	C	A	28.03	LowQual; QDFilter	0.85	5,28,0,0	32	G182V	NS_CODING	ASPH	ENST00000518306
chr18	31325901	C	T	12.62	HaplotypeFilter; LowQual; QDFilter	0.49	0,19,0,7	26	A2030V	NS_CODING	ASXL3	ENST00000269197
chr19	50435934	T	C	47.13	HaplotypeFilter; QDFilter	0.58	0,22,0,58	80	L145P	NS_CODING	ATF5	ENST00000423777
chr19	50435939	T	C	72.31	HaplotypeFilter; QDFilter	0.86	0,22,1,61	84	S147P	NS_CODING	ATF5	ENST00000423777
chrX	152845631	A	C	12.63	HaplotypeFilter; LowQual; QDFilter	0.1	89,34,1,1	123	N1180H	NS_CODING	ATP2B3	ENST00000263519
chr12	111993712	C	T	84.11	LowCovFilter	28.04	0,0,0,3	3	S248N	NS_CODING	ATXN2	ENST00000377617
chr11	134253627	C	T	169.32	PASS	2.2	0,65,0,12	77	V190M	NS_CODING	B3GAT1	ENST00000312527
chr8	143592303	T	C	22.56	LowCovFilter; LowQual; QDFilter	1.41	0,4,0,12	16	S896P	NS_CODING	BAI1	ENST00000323289
chr8	143592306	G	C	32.5	LowCovFilter	2.03	0,5,10,1	16	A897P	NS_CODING	BAI1	ENST00000323289
chr6	136582537	G	A	362.56	QDFilter	1.28	35,0,248,0	280	R702C	NS_CODING	BCLAF1	ENST00000530767
chr19	15353818	T	G	89.77	HaplotypeFilter	2.64	0,0,11,22	33	H1021P	NS_CODING	BRD4	ENST00000263377
chr19	15355273	A	G	12.84	HaplotypeFilter; LowQual; QDFilter	0.18	53,1,17,1	70	S784P	NS_CODING	BRD4	ENST00000263377
chr12	40085906	A	T	122.27	LowCovFilter	30.57	0,0,0,4	4	K468M	NS_CODING	C12orf40	ENST00000324616
chr19	38795587	A	G	614.39	ReadPosFilter	2.38	217,0,41,0	256	K102E	NS_CODING	C19orf33	ENST00000301246
chr19	38795590	G	A	376.85	QDFilter; ReadPosFilter	1.48	36,0,218,0	252	E103K	NS_CODING	C19orf33	ENST00000301246
chr6	42075097	T	G	57.59	HaplotypeFilter; QDFilter	1.4	0,0,10,31	41	S185R	NS_CODING	C6orf132	ENST00000341865

Table S4: Novel SNPs found in genes of MOLM-13 resistant cells (continued, Page 4)

CHR	POSITION	REF BASE	OBS BASE	QUALITY SCORE	FILTER	QD	A,C,G,T	COV	AA CHANGE	EFFECT	GENE NAME	TRANSCRIPT ID
chr15	44671983	C	A	11.68	LowQual; QDFilter	0.56	4,17,0,0	21	P279T	NS_CODING	CASC4	ENST00000416522
chr5	95865526	G	A	74.28	LowCovFilter	14.86	3,0,2,0	5	G1E	NS_CODING	CAST	ENST00000505143
chr17	20768744	G	T	126.23	QDFilter	1.34	0,0,73,21	94	S217Y	NS_CODING	CCDC144NL	ENST00000327925
chr17	20768788	G	T	13.78	LowQual; QDFilter	0.12	0,0,100,18	116	H202Q	NS_CODING	CCDC144NL	ENST00000327925
chr17	20768816	C	T	128.48	QDFilter	1.16	0,92,0,19	109	C193Y	NS_CODING	CCDC144NL	ENST00000327925
chr17	20768730	A	G	117.95	QDFilter	1.47	63,0,17,0	80	*222Q	STOP_LOST	CCDC144NL	ENST00000327925
chr2	132288362	T	C	54.81	QDFilter	0.75	0,16,0,57	72	M103T	NS_CODING	CCDC74A	ENST00000409856
chr2	130897620	A	G	113.71	PASS	2.71	32,0,10,0	42	S207P	NS_CODING	CCDC74B	ENST00000409488
chr20	23066480	A	C	45.91	FSFilter; QDFilter	0.45	76,26,0,1	102	V117G	NS_CODING	CD93	ENST00000246006
chr17	45234657	T	C	59.73	PASS	2.6	0,6,0,17	21	N129S	NS_CODING	CDC27	ENST00000446365
chr17	45234707	T	A	43.31	QDFilter	1.24	6,0,0,29	31	L112F	NS_CODING	CDC27	ENST00000446365
chr1	22304428	C	G	22.91	LowQual; QDFilter	0.57	0,35,5,0	39	P14A	NS_CODING	CELA3B	ENST00000374666
chr1	22304429	C	T	14.05	LowQual; QDFilter	0.33	0,38,0,5	42	P14L	NS_CODING	CELA3B	ENST00000374666
chr14	81362120	G	T	12.29	LowCovFilter; LowQual; QDFilter	0.95	0,0,9,4	13	T136K	NS_CODING	CEP128	ENST00000327841
chr1	243328887	G	T	91.69	FSFilter; QDFilter	0.47	0,0,176,20	196	S694*	STOP_GAINED	CEP170	ENST00000366543
chr1	196759282	C	T	35.63	LowCovFilter	17.82	0,0,0,2	2	P180S	NS_CODING	CFHR3	ENST00000391985
chr15	78873177	C	A	61.2	PASS	2.55	4,20,0,0	23	A44E	NS_CODING	CHRNA5	ENST00000299565
chr8	68334756	G	T	352.28	PASS	2.3	0,0,131,22	150	L189I	NS_CODING	CPA6	ENST00000297769
chr21	44589932	A	C	30.26	LowCovFilter; QDFilter	1.89	11,5,0,0	16	H33P	NS_CODING	CRYAA	ENST00000398133
chr8	41132742	A	T	52.75	QDFilter	1.65	27,0,0,5	32	S43T	NS_CODING	CTD-3080F16.3	ENST00000517495
chr22	42523636	C	A	187.92	FSFilter;QDFilter	0.46	49,359,0,0	408	R275L	NS_CODING	CYP2D6	ENST00000413640

Table S4: Novel SNPs found in genes of MOLM-13 resistant cells (continued, Page 5)

CHR	POSITION	REF BASE	OBS BASE	QUALITY SCORE	FILTER	QD	A,C,G,T	COV	AA CHANGE	EFFECT	GENE NAME	TRANSCRIPT ID
chr19	15989696	G	C	35.61	QDFilter	0.32	0,15,98,0	111	A334G	NS_CODING	CYP4F2	ENST00000392846
chr22	24180858	T	G	30.96	HaplotypeFilter; QDFilter	0.35	0,0,26,63	89	T47P	NS_CODING	DERL3	ENST00000404056
chr10	124356559	C	G	21.86	LowCovFilter; LowQual	3.64	0,4,2,0	6	T470R	NS_CODING	DMBT1	ENST00000327438
chr19	36002380	C	T	34.2	LowCovFilter	2.28	0,12,0,3	15	G284D	NS_CODING	DMKN	ENST00000339686
chr19	36002385	A	T	43.25	HaplotypeFilter; LowCovFilter	3.93	8,0,0,3	11	S282R	NS_CODING	DMKN	ENST00000339686
chr19	36002393	C	T	21.86	HaplotypeFilter; LowCovFilter; LowQual	2.43	0,6,0,3	9	G280S	NS_CODING	DMKN	ENST00000339686
chr19	36002399	T	C	18.88	LowCovFilter; LowQual	2.1	0,3,0,6	9	S278G	NS_CODING	DMKN	ENST00000339686
chr13	50242538	T	A	46.21	LowCovFilter	9.24	3,0,0,2	5	T137S	NS_CODING	EBPL	ENST00000378268
chr1	245133593	T	G	83.64	HaplotypeFilter; QDFilter	1.12	0,1,16,57	74	S57A	NS_CODING	EFCAB2	ENST00000366522
chr11	65359245	G	C	10.73	LowCovFilter; LowQual; QDFilter	0.89	0,2,10,0	12	A25P	NS_CODING	EHBP1L1	ENST00000533364
chr2	119604535	T	G	35.97	LowCovFilter	2.57	0,0,8,6	14	H70P	NS_CODING	EN1	ENST00000295206
chr12	132445261	A	C	19.21	LowQual; QDFilter	0.77	16,9,0,0	25	N33H	NS_CODING	EP400	ENST00000330386
chr1	16386495	C	T	70.66	LowCovFilter	6.42	0,7,0,4	11	R107Q	NS_CODING	FAM131C	ENST00000375662
chr11	6238961	G	C	105.87	HaplotypeFilter; QDFilter	0.88	0,36,83,0	118	R544G	NS_CODING	FAM160A2	ENST00000442917
chr20	26061956	C	T	96.35	PASS	2.83	4,23,0,7	34	A103V	NS_CODING	FAM182A	ENST00000246000
chr20	26062003	A	T	37.26	LowCovFilter	2.87	10,0,0,3	13	K119*	STOP_GAINED	FAM182A	ENST00000246000
chr20	26061865	G	T	23.05	LowQual;QDFilter	0.48	0,0,40,8	48	E14*	STOP_GAINED	FAM182A	ENST00000415411
chr20	25755526	C	T	41.02	QDFilter	1.86	0,17,0,5	21	G144R	NS_CODING	FAM182B	ENST00000376403
chr20	25755558	G	T	37.32	LowCovFilter	2.67	0,0,10,4	14	S133Y	NS_CODING	FAM182B	ENST00000376403

Table S4: Novel SNPs found in genes of MOLM-13 resistant cells (continued, Page 6)

CHR	POSITION	REF BASE	OBS BASE	QUALITY SCORE	FILTER	QD	A,C,G,T	COV	AA CHANGE	EFFECT	GENE NAME	TRANSCRIPT ID
chr20	25755583	C	T	16.04	LowCovFilter; LowQual; QDFilter	1.46	1,7,0,3	11	D125N	NS_CODING	FAM182B	ENST00000376403
chr20	25755585	C	T	10.33	LowCovFilter; LowQual; QDFilter	0.86	0,9,0,3	12	S124N	NS_CODING	FAM182B	ENST00000376403
chr7	102417753	T	G	63.28	LowCovFilter	4.22	0,0,5,10	15	Y180D	NS_CODING	FAM185A	ENST00000409231
chr11	71498601	G	T	35.61	LowCovFilter	7.12	0,0,3,2	5	A7S	NS_CODING	FAM86C1	ENST00000346333
chr11	71498671	G	C	20.46	LowCovFilter; LowQual	3.41	0,2,4,0	6	R30P	NS_CODING	FAM86C1	ENST00000346333
chrX	54497142	T	C	24.59	HaplotypeFilter; LowQual; QDFilter	1.02	0,8,0,15	23	E178G	NS_CODING	FGD1	ENST00000375135
chr1	152280665	G	A	451.89	QDFilter	1.14	55,1,339,0	394	P2233S	NS_CODING	FLG	ENST00000368799
chr1	152280671	A	C	210.94	QDFilter	0.53	349,47,0,0	395	S2231A	NS_CODING	FLG	ENST00000368799
chr1	152280685	C	A	371.82	FSFilter; QDFilter	0.87	57,372,0,0	428	G2226V	NS_CODING	FLG	ENST00000368799
chr1	152280688	A	G	369.13	FSFilter; QDFilter	0.84	380,0,57,0	436	V2225A	NS_CODING	FLG	ENST00000368799
chr1	152281534	A	T	254.59	QDFilter	1.25	179,0,0,25	204	L1943H	NS_CODING	FLG	ENST00000368799
chr1	152280670	G	T	204.19	QDFilter	0.52	0,1,347,46	393	S2231*	STOP_GAINED	FLG	ENST00000368799
chr13	28592642	C	A	938.47	PASS	9.02	37,67,0,0	103	D835Y	NS_CODING	FLT3	ENST00000241453
chr1	240370952	C	T	58.96	PASS	2.11	0,23,0,5	28	P947L	NS_CODING	FMN2	ENST00000319653
chr14	44974189	C	A	140.58	LowCovFilter	14.06	5,5,0,0	10	A561S	NS_CODING	FSCB	ENST00000537803
chr16	81129822	G	A	55.43	LowCovFilter	7.92	3,0,4,0	7	S21L	NS_CODING	GCSH	ENST00000315467
chr19	48197565	A	C	112.02	HaplotypeFilter; QDFilter	1.75	37,27,0,0	64	Q826P	NS_CODING	GLTSCR1	ENST00000396720
chr9	36216341	T	C	18.05	LowCovFilter; LowQual; QDFilter	0.9	0,4,0,16	20	T95A	NS_CODING	GNE	ENST00000486079

Table S4: Novel SNPs found in genes of MOLM-13 resistant cells (continued, Page 7)

CHR	POSITION	REF BASE	OBS BASE	QUALITY SCORE	FILTER	QD	A,C,G,T	COV	AA CHANGE	EFFECT	GENE NAME	TRANSCRIPT ID
chr15	23685002	T	C	80.61	HaplotypeFilter; QDFilter	0.68	0,14,0,105	118	R279G	NS_CODING	GOLGA6L2	ENST00000345070
chr15	23686019	G	C	57.2	HaplotypeFilter; QDFilter	0.21	1,27,244,1	273	R262G	NS_CODING	GOLGA6L2	ENST00000345070
chr1	6310024	C	T	35.08	LowCovFilter	8.77	0,2,0,2	4	V402M	NS_CODING	GPR153	ENST00000377893
chr17	36484681	C	A	4120.41	PASS	15.15	140,132,0,0	269	E1591*	STOP_GAINED	GPR179	ENST00000342292
chr11	105481617	G	T	12.29	LowCovFilter; LowQual; QDFilter	1.76	0,0,5,2	7	NA	START_GAINED	GRIA4	ENST00000428631
chr1	110231874	G	T	10.43	LowCovFilter; LowQual	10.43	0,0,0,1	1	R115L	NS_CODING	GSTM1	ENST00000369823
chr6	29910557	T	A	64.46	LowCovFilter	9.21	7,0,0,0	7	F33I	NS_CODING	HLA-A	ENST00000355767
chr6	29910558	T	C	64.46	LowCovFilter	9.21	0,7,0,0	7	F33S	NS_CODING	HLA-A	ENST00000355767
chr6	31324100	G	T	10.43	LowCovFilter; LowQual	3.48	0,0,0,3	3	R155S	NS_CODING	HLA-B	ENST00000412585
chr6	32489881	G	C	54.17	LowCovFilter	13.54	0,4,0,0	4	H57Q	NS_CODING	HLA-DRB5	ENST00000374975
chr15	65688593	T	C	59.26	QDFilter	1.21	0,9,0,40	49	E32G	NS_CODING	IGDCC4	ENST00000356152
chr15	65688597	T	C	11.15	LowQual; QDFilter	0.23	0,6,0,43	49	R31G	NS_CODING	IGDCC4	ENST00000356152
chr2	217498311	T	C	33.74	LowCovFilter	11.25	0,2,0,1	3	L22P	NS_CODING	IGFBP2	ENST00000233809
chr1	201178736	C	T	79.16	QDFilter	0.22	1,319,1,41	355	A1572V	NS_CODING	IGFN1	ENST00000335211
chr1	201178904	A	G	79.51	QDFilter	0.49	139,0,21,1	160	E1628G	NS_CODING	IGFN1	ENST00000335211
chr15	41275974	G	A	124.29	LowCovFilter	8.88	6,0,8,0	14	S1408L	NS_CODING	INO80	ENST00000361937
chr19	11283661	C	A	1197.67	PASS	7.93	46,105,0,0	150	S736I	NS_CODING	KANK2	ENST00000355150
chr7	119915308	C	A	36.91	QDFilter	0.24	15,138,0,0	152	P208T	NS_CODING	KCND2	ENST00000331113
chr17	7751162	T	C	13.99	LowQual; QDFilter	0.47	0,7,0,23	30	L519P	NS_CODING	KDM6B	ENST00000254846
chr6	96972281	C	A	104.69	LowCovFilter	8.72	5,7,0,0	12	Q83K	NS_CODING	KIAA0776	ENST00000369278
chr19	55328993	G	C	10.43	LowCovFilter; LowQual;QDFilter	0.95	0,11,0,0	11	L13F	NS_CODING	KIR3DL1	ENST00000326542

Table S4: Novel SNPs found in genes of MOLM-13 resistant cells (continued, Page 8)

CHR	POSITION	REF BASE	OBS BASE	QUALITY SCORE	FILTER	QD	A,C,G,T	COV	AA CHANGE	EFFECT	GENE NAME	TRANSCRIPT ID
chr12	10571091	T	G	127.61	LowCovFilter	31.9	0,0,4,0	4	H113P	NS_CODING	KLRC3	ENST00000381903
chr17	38975100	T	C	95.44	QDFilter	0.73	0,18,0,112	130	S563G	NS_CODING	KRT10	ENST00000269576
chr17	38975162	T	G	152.22	HaplotypeFilter	2.62	1,4,10,43	58	Y542S	NS_CODING	KRT10	ENST00000269576
chr17	38975166	C	T	93.05	HaplotypeFilter; QDFilter	1.6	0,48,0,8	56	G541S	NS_CODING	KRT10	ENST00000269576
chr17	39595484	G	A	21.36	LowQual; QDFilter	0.34	9,0,53,0	62	Q235*	STOP_GAINED	KRT38	ENST00000246646
chr17	39197549	G	C	160.6	QDFilter	1.18	0,20,116,0	134	S34C	NS_CODING	KRTAP1-1	ENST00000306271
chr11	1605983	G	C	26.23	LowQual; QDFilter	1.01	0,6,20,0	26	A166G	NS_CODING	KRTAP5-1	ENST00000382171
chr11	71279936	A	C	68.83	QDFilter	1	58,11,0,0	69	K157Q	NS_CODING	KRTAP5-10	ENST00000376536
chr11	1651615	A	G	54.97	QDFilter	0.43	118,0,11,0	129	Y153C	NS_CODING	KRTAP5-5	ENST00000422553
chr10	88476146	T	C	69.99	HaplotypeFilter	3.04	1,14,0,8	23	S175P	NS_CODING	LDB3	ENST00000352360
chr10	88476152	G	C	72.89	HaplotypeFilter	2.92	0,11,11,3	25	A177P	NS_CODING	LDB3	ENST00000352360
chr10	88476158	G	C	29.55	HaplotypeFilter; LowQual; QDFilter	1.28	0,9,13,0	22	A179P	NS_CODING	LDB3	ENST00000352360
chr10	88476170	T	C	198.37	HaplotypeFilter	7.93	0,17,0,8	25	S183P	NS_CODING	LDB3	ENST00000352360
chr10	88476176	G	C	119.81	PASS	4.44	1,15,11,0	27	A185P	NS_CODING	LDB3	ENST00000352360
chr1	226075558	C	T	164.93	LowCovFilter	11	0,6,0,9	15	R142Q	NS_CODING	LEFTY1	ENST00000272134
chr19	54726861	C	T	80.87	QDFilter	0.56	0,127,0,18	142	NA	START_GAINED	LILRB3	ENST00000346401
chr15	34659253	C	G	63.76	HaplotypeFilter; QDFilter	0.9	1,53,17,0	70	G17R	NS_CODING	LPCAT4	ENST00000314891
chr19	45649085	T	C	28.19	LowCovFilter; LowQual; QDFilter	1.76	0,5,0,11	16	S587P	NS_CODING	LRRC68	ENST00000421905
chr8	92136728	C	T	204.37	PASS	5.84	0,25,0,10	34	T64I	NS_CODING	LRRC69	ENST00000448384
chrX	140993642	C	G	127.43	QDFilter	1.52	0,71,13,0	84	T151S	NS_CODING	MAGEC1	ENST00000285879
chrX	140993649	T	A	136.48	QDFilter	1.68	13,0,0,68	81	S153R	NS_CODING	MAGEC1	ENST00000285879

Table S4: Novel SNPs found in genes of MOLM-13 resistant cells (continued, Page 9)

CHR	POSITION	REF BASE	OBS BASE	QUALITY SCORE	FILTER	QD	A,C,G,T	COV	AA CHANGE	EFFECT	GENE NAME	TRANSCRIPT ID
chr22	40814743	C	G	34.83	QDFilter	1.09	0,19,13,0	30	A517P	NS_CODING	MKL1	ENST00000402042
chr7	151945334	T	C	201.67	QDFilter	1.45	0,40,0,99	138	N729D	NS_CODING	MLL3	ENST00000262189
chr15	56723662	C	A	14.55	LowCovFilter; LowQual; QDFilter	0.77	4,15,0,0	19	R435M	NS_CODING	MNS1	ENST00000260453
chr22	31334052	A	G	2395.73	PASS	16.99	63,0,78,0	140	M343T	NS_CODING	MORC2	ENST00000215862
chr1	17083888	C	T	72.11	PASS	3	0,19,0,5	23	G637S	NS_CODING	MST1P9	ENST00000334998
chr1	17086941	C	T	135.19	PASS	3.38	0,32,0,8	40	R128H	NS_CODING	MST1P9	ENST00000334998
chr19	9002623	C	T	97.96	QDFilter	0.64	0,136,0,16	151	R13398H	NS_CODING	MUC16	ENST00000397910
chr11	1016640	G	A	437.42	HaplotypeFilter; QDFilter	0.86	61,0,431,18	508	A2054V	NS_CODING	MUC6	ENST00000421673
chr11	1017280	G	T	385	HaplotypeFilter; QDFilter	0.58	0,0,589,78	665	P1841T	NS_CODING	MUC6	ENST00000421673
chr11	1017294	A	T	217.91	HaplotypeFilter; QDFilter	0.27	730,0,1,85	812	L1836H	NS_CODING	MUC6	ENST00000421673
chr11	1017307	G	A	547.68	HaplotypeFilter; QDFilter	0.6	118,0,792,1	907	P1832S	NS_CODING	MUC6	ENST00000421673
chr11	1018024	C	G	205.39	HaplotypeFilter; QDFilter	0.56	0,332,35,1	366	A1593P	NS_CODING	MUC6	ENST00000421673
chr11	1018262	G	C	447.66	HaplotypeFilter; QDFilter	1.57	0,59,206,7	270	H1513Q	NS_CODING	MUC6	ENST00000421673
chr18	9124964	A	G	88.36	LowCovFilter	4.65	15,0,4,0	19	I188V	NS_CODING	NDUFV2	ENST00000318388
chr1	120611554	T	C	143.38	QDFilter	0.49	0,37,0,255	292	N172S	NS_CODING	NOTCH2	ENST00000538680
chr1	120611555	T	G	116.24	QDFilter	0.39	0,1,36,258	295	N172H	NS_CODING	NOTCH2	ENST00000538680
chr11	17351683	C	G	77.89	LowCovFilter	12.98	0,3,3,0	6	Q308E	NS_CODING	NUCB2	ENST00000458064
chr11	48346535	G	C	269.32	PASS	3.79	0,15,56,0	71	G15R	NS_CODING	OR4C3	ENST00000319856
chr11	48346541	A	T	255.01	PASS	3.4	60,0,0,15	75	T17S	NS_CODING	OR4C3	ENST00000319856
chr11	48387946	G	T	42.83	QDFilter	0.56	0,0,65,12	77	F24L	NS_CODING	OR4C5	ENST00000319813
chr11	4976241	C	G	15.29	LowQual; QDFilter	0.14	0,91,15,0	103	E235Q	NS_CODING	OR51A2	ENST00000380371

Table S4: Novel SNPs found in genes of MOLM-13 resistant cells (continued, Page 10)

CHR	POSITION	REF BASE	OBS BASE	QUALITY SCORE	FILTER	QD	A,C,G,T	COV	AA CHANGE	EFFECT	GENE NAME	TRANSCRIPT ID
chr11	4967831	T	C	470.79	PASS	2.39	0,38,0,159	195	N167S	NS_CODING	OR51A4	ENST00000380373
chr11	124267148	C	T	40.27	QDFilter	0.56	0,57,0,15	71	V34I	NS_CODING	OR8B3	ENST00000354597
chr11	55873210	C	A	1173.96	PASS	13.04	41,49,0,0	89	T231N	NS_CODING	OR8H2	ENST00000313503
chr11	56143370	G	A	80.24	QDFilter	0.74	14,0,93,1	107	V91I	NS_CODING	OR8U1	ENST00000302270
chr11	56143371	T	G	80.22	QDFilter	0.75	0,0,14,93	106	V91G	NS_CODING	OR8U1	ENST00000302270
chr11	56143382	G	A	69.23	QDFilter	0.62	13,0,98,0	110	D95N	NS_CODING	OR8U1	ENST00000302270
chr11	56143394	A	G	210.3	QDFilter	1.7	106,0,18,0	123	T99A	NS_CODING	OR8U1	ENST00000302270
chr11	56143415	A	G	349.59	PASS	2.59	110,0,24,1	133	T106A	NS_CODING	OR8U1	ENST00000302270
chr14	20919551	C	T	88.62	LowCovFilter	29.54	0,0,0,3	3	G158R	NS_CODING	OSGEP	ENST00000488532
chr8	101719004	G	A	278.37	QDFilter	1.61	33,0,140,0	169	R2C	NS_CODING	PABPC1	ENST00000517990
chr13	25051938	A	G	28.25	LowQual; QDFilter	1.35	18,0,3,0	21	F564L	NS_CODING	PARP4	ENST00000381989
chr13	25051939	T	G	33.78	LowCovFilter; QDFilter	1.69	0,0,3,17	20	K563N	NS_CODING	PARP4	ENST00000381989
chrY	4968655	T	G	58.28	LowCovFilter	3.64	0,0,15,1	16	N1001K	NS_CODING	PCDH11Y	ENST00000333703
chr5	140558212	A	C	55.76	LowCovFilter	9.29	3,3,0,0	6	K199N	NS_CODING	PCDHB8	ENST00000239444
chr12	123474405	T	G	15.93	LowCovFilter; LowQual; QDFilter	1.77	0,0,2,7	9	H608P	NS_CODING	PITPNM2	ENST00000392428
chr11	64032523	G	C	47.19	HaplotypeFilter; QDFilter	1.43	0,9,24,0	33	R851P	NS_CODING	PLCB3	ENST00000325234
chr3	145803142	T	A	199.33	LowCovFilter	33.22	6,0,0,0	6	K9I	NS_CODING	PLOD2	ENST00000461497
chr12	106820975	C	T	10.42	HaplotypeFilter; LowCovFilter; LowQual; QDFilter	0.52	0,9,0,11	20	L126F	NS_CODING	POLR3B	ENST00000549569
chr7	72396209	T	C	41.88	HaplotypeFilter; QDFilter	1.16	0,13,0,22	35	S184P	NS_CODING	POM121	ENST00000434423
chr12	3649792	G	C	62.01	HaplotypeFilter; QDFilter	0.76	6,25,50,0	79	Q23H	NS_CODING	PRMT8	ENST00000452611

Table S4: Novel SNPs found in genes of MOLM-13 resistant cells (continued, Page 11)

CHR	POSITION	REF BASE	OBS BASE	QUALITY SCORE	FILTER	QD	A,C,G,T	COV	AA CHANGE	EFFECT	GENE NAME	TRANSCRIPT ID
chr12	3649800	A	C	58.22	HaplotypeFilter; QDFilter	0.75	55,22,1,0	76	Q26P	NS_CODING	PRMT8	ENST00000452611
chr2	240982075	A	G	10.22	LowCovFilter; LowQual; QDFilter	0.85	9,0,3,0	12	C109R	NS_CODING	PRR21	ENST00000408934
chr2	240982129	G	C	50.19	QDFilter	1.79	0,6,22,0	28	P91A	NS_CODING	PRR21	ENST00000408934
chr2	240982131	A	G	80.65	PASS	2.88	22,0,6,0	28	M90T	NS_CODING	PRR21	ENST00000408934
chr2	240982144	T	C	77.63	PASS	2.59	0,6,1,23	30	S86G	NS_CODING	PRR21	ENST00000408934
chr7	142458929	G	C	54.44	QDFilter	1.21	0,9,36,0	43	K70N	NS_CODING	PRSS1	ENST00000486171
chr7	142458938	T	A	33.06	QDFilter	0.85	8,0,0,31	38	F73L	NS_CODING	PRSS1	ENST00000486171
chr9	33798574	G	A	121.92	QDFilter	0.63	32,0,163,0	193	S175N	NS_CODING	PRSS3	ENST00000429677
chr2	204305595	A	G	73.52	HaplotypeFilter; QDFilter	0.78	63,0,31,0	94	L773P	NS_CODING	RAPH1	ENST00000319170
chr7	102246311	T	C	49.06	LowCovFilter	8.18	0,3,0,3	6	E122G	NS_CODING	RASA4	ENST00000522801
chr19	1848243	A	G	1000.23	PASS	38.47	0,0,26,0	26	S39P	NS_CODING	REXO1	ENST00000170168
chr8	54870974	A	C	112.13	HaplotypeFilter; QDFilter	0.87	105,24,0,0	127	K109Q	NS_CODING	RGS20	ENST00000522225
chr9	34725432	C	T	35.63	LowCovFilter	2.74	0,3,0,10	13	R602H	NS_CODING	RP11-195F19.10	ENST00000378788
chr9	34725438	A	G	35.63	LowCovFilter	2.55	0,0,14,0	14	L600S	NS_CODING	RP11-195F19.10	ENST00000378788
chr20	17639850	C	T	32.69	HaplotypeFilter; QDFilter	0.09	0,337,1,39	376	A435T	NS_CODING	RRBP1	ENST00000246043
chr20	17639876	T	G	84.96	HaplotypeFilter; QDFilter	0.33	1,2,40,217	259	Q426P	NS_CODING	RRBP1	ENST00000246043
chr15	77176200	A	T	127.05	LowCovFilter	12.71	5,0,0,5	10	V41D	NS_CODING	SCAPER	ENST00000303521
chr7	3341390	T	C	11.01	LowCovFilter; LowQual	11.01	0,1,0,0	1	S58P	NS_CODING	SDK1	ENST00000389531
chr10	102269085	C	A	434.18	PASS	18.88	14,9,0,0	23	L129F	NS_CODING	SEC31B	ENST00000370329
chr12	109017650	G	C	175.13	QDFilter	0.89	0,24,172,1	195	P135R	NS_CODING	SELPLG	ENST00000388962
chr2	110371493	A	T	47.98	LowCovFilter	23.99	0,0,0,2	2	NA	START_GAINED	SEPT10	ENST00000352314

Table S4: Novel SNPs found in genes of MOLM-13 resistant cells (continued, Page 12)

CHR	POSITION	REF BASE	OBS BASE	QUALITY SCORE	FILTER	QD	A,C,G,T	COV	AA CHANGE	EFFECT	GENE NAME	TRANSCRIPT ID
chr6	146234615	T	A	25.95	LowCovFilter; LowQual	2.59	2,0,0,8	9	Q1442L	NS_CODING	SHPRH	ENST00000275233
chr19	51920196	G	T	109.5	QDFilter	0.45	0,0,217,25	240	Q111K	NS_CODING	SIGLEC10	ENST00000530476
chr20	1895965	C	A	635.42	LowCovFilter	30.26	20,0,0,0	20	N100K	NS_CODING	SIRPA	ENST00000356025
chr1	1599812	C	T	10.6	LowQual; QDFilter	0.5	0,18,0,3	21	V312I	NS_CODING	SLC35E2B	ENST00000234800
chr13	88325757	A	G	22.84	HaplotypeFilter; LowQual; QDFilter	0.36	43,0,20,0	63	E8G	NS_CODING	SLITRK5	ENST00000400028
chr2	242011126	A	C	124.09	HaplotypeFilter; QDFilter	1.28	57,38,1,1	96	Q1242P	NS_CODING	SNED1	ENST00000310397
chr15	45364534	A	C	35.63	LowCovFilter	17.82	0,2,0,0	2	N269T	NS_CODING	SORD	ENST00000267814
chr2	231248261	C	T	144.95	LowCovFilter	13.18	0,4,0,7	11	T225M	NS_CODING	SP140L	ENST00000243810
chr8	145095687	T	C	834.79	FSFilter; HaplotypeFilter	2.2	3,116,1,259	377	S329P	NS_CODING	SPATC1	ENST00000377470
chr1	16262471	A	C	58.99	HaplotypeFilter; QDFilter	1.55	24,14,0,0	37	T3246P	NS_CODING	SPEN	ENST00000375759
chr1	16262483	G	C	24.32	HaplotypeFilter; LowQual; QDFilter	0.58	0,11,29,1	40	V3250L	NS_CODING	SPEN	ENST00000375759
chr1	16262484	T	C	48.12	HaplotypeFilter; QDFilter	1.07	1,8,1,33	42	V3250A	NS_CODING	SPEN	ENST00000375759
chr1	206566903	G	A	189.21	HaplotypeFilter; QDFilter	1.31	29,0,114,0	141	R149H	NS_CODING	SRGAP2	ENST00000414359
chr19	56029556	C	G	216.36	HaplotypeFilter; QDFilter	0.69	0,271,39,4	313	P1305A	NS_CODING	SSC5D	ENST00000389623
chr18	55019921	A	C	58.29	LowCovFilter	11.66	1,4,0,0	5	H56P	NS_CODING	ST8SIA3	ENST00000541833
chr18	55019927	G	C	88.73	LowCovFilter	17.75	0,4,1,0	5	R58P	NS_CODING	ST8SIA3	ENST00000541833
chr10	104263946	A	C	11.51	HaplotypeFilter; LowQual; QDFilter	0.28	24,17,0,0	41	T13P	NS_CODING	SUFU	ENST00000369899

Table S4: Novel SNPs found in genes of MOLM-13 resistant cells (continued, Page 13)

CHR	POSITION	REF BASE	OBS BASE	QUALITY SCORE	FILTER	QD	A,C,G,T	COV	AA CHANGE	EFFECT	GENE NAME	TRANSCRIPT ID
chr7	855880	T	C	18.86	LowCovFilter; LowQual	3.77	0,2,0,3	5	I16T	NS_CODING	SUN1	ENST00000456758
chr7	105733100	C	A	12.36	LowCovFilter; LowQual	6.18	2,0,0,0	2	V203F	NS_CODING	SYPL1	ENST00000470347
chr1	47685603	A	C	37.54	QDFilter	1.5	18,7,0,0	25	V262G	NS_CODING	TAL1	ENST00000294339
chr12	11244687	G	C	157.79	PASS	2.77	0,14,43,0	57	L48V	NS_CODING	TAS2R43	ENST00000531678
chr6	167592571	G	A	56.73	QDFilter	0.59	13,0,83,0	95	A244T	NS_CODING	TCP10L2	ENST00000366832
chr16	4310221	T	G	62.14	QDFilter	1.55	0,0,13,27	40	H231P	NS_CODING	TFAP4	ENST00000204517
chr19	48305574	A	G	19.6	HaplotypeFilter; LowQual; QDFilter	0.25	65,0,14,0	78	S222P	NS_CODING	TPRX1	ENST00000543508
chr3	14106295	C	T	85.96	PASS	3.74	0,18,0,5	23	P207S	NS_CODING	TPRXL	ENST00000326972
chr3	14106302	G	C	151	PASS	6.04	0,7,18,0	25	S209T	NS_CODING	TPRXL	ENST00000326972
chr16	1291598	G	A	108.44	QDFilter	1.64	19,0,47,0	65	V133I	NS_CODING	TPSAB1	ENST00000338844
chr16	1291608	A	G	84.38	QDFilter	1.14	56,1,17,0	73	H136R	NS_CODING	TPSAB1	ENST00000338844
chr16	1307060	A	G	52.47	QDFilter	0.52	89,1,11,0	99	N166D	NS_CODING	TPSD1	ENST00000397534
chr7	142181009	G	C	44.86	QDFilter	0.69	0,9,56,0	65	NA	START_GAINED	TRBV6-5	ENST00000390368
chr11	5663706	G	C	245.66	PASS	11.7	0,9,12,0	21	D282H	NS_CODING	TRIM34	ENST00000429814
chr18	72997831	A	C	62.21	HaplotypeFilter; QDFilter	1.22	38,13,0,0	51	T112P	NS_CODING	TSHZ1	ENST00000322038
chr6	3154989	G	C	35.63	LowCovFilter	17.82	0,2,0,0	2	T149S	NS_CODING	TUBB2A	ENST00000333628
chr6	3154996	T	G	35.63	LowCovFilter	17.82	0,0,2,0	2	M147L	NS_CODING	TUBB2A	ENST00000333628
chr6	3154999	C	A	35.63	LowCovFilter	17.82	2,0,0,0	2	G146W	NS_CODING	TUBB2A	ENST00000333628
chrX	153151280	G	C	11.76	LowCovFilter; LowQual; QDFilter	0.78	0,4,11,0	15	A49P	NS_CODING	U52112.12	ENST00000357566
chr2	181854692	C	T	53.39	LowCovFilter	5.93	0,5,0,4	9	S110L	NS_CODING	UBE2E3	ENST00000409513
chr11	5536663	C	A	1124.24	PASS	12.49	40,49,1,0	90	G337C	NS_CODING	UBQLNL	ENST00000380184
chr9	134406650	C	T	45.05	LowCovFilter	22.53	0,0,0,2	2	NA	START_GAINED	UCK1	ENST00000372215

Table S4: Novel SNPs found in genes of MOLM-13 resistant cells (continued, Page 14)

CHR	POSITION	REF BASE	OBS BASE	QUALITY SCORE	FILTER	QD	A,C,G,T	COV	AA CHANGE	EFFECT	GENE NAME	TRANSCRIPT ID
chr4	115544281	C	A	588.65	PASS	5.21	27,86,0,0	112	S82*	STOP_GAINED	UGT8	ENST00000310836
chr9	35386199	T	C	879.83	PASS	12.05	0,32,0,41	72	V506A	NS_CODING	UNC13B	ENST00000535471
chr4	96256628	G	T	474.84	PASS	9.89	0,0,30,18	46	F52L	NS_CODING	UNC5C	ENST00000331502
chr10	118897591	G	A	16.23	LowQual; QDFilter	0.34	13,0,35,0	48	NA	START_GAINED	VAX1	ENST00000277905
chr5	82837439	G	T	477.17	PASS	6.2	0,0,56,21	76	E1886*	STOP_GAINED	VCAN	ENST00000343200
chr3	11606445	G	T	1141.85	PASS	14.83	0,0,37,40	77	S101R	NS_CODING	VGLL4	ENST00000273038
chr7	12400783	C	T	30.52	LowCovFilter	6.1	0,3,0,2	5	C1050Y	NS_CODING	VWDE	ENST00000275358
chr15	83481859	T	A	29.76	LowCovFilter; LowQual	4.25	3,0,0,4	6	I205N	NS_CODING	WHAMM	ENST00000234505
chr12	65477602	C	T	43.23	LowCovFilter	3.6	0,9,0,3	11	R11K	NS_CODING	WIF1	ENST00000535025
chr7	29923888	A	C	215.88	HaplotypeFilter	4.69	28,17,0,0	45	I260L	NS_CODING	WIPF3	ENST00000242140
chr7	29923901	T	C	35.64	QDFilter	0.91	0,14,0,25	39	L264P	NS_CODING	WIPF3	ENST00000242140
chr7	100349919	T	C	147.34	HaplotypeFilter; QDFilter	0.92	4,26,0,130	157	S731P	NS_CODING	ZAN	ENST00000538115
chr19	58420682	T	C	171.69	QDFilter	0.37	0,65,0,395	454	R123G	NS_CODING	ZNF417	ENST00000536263
chr19	58003359	T	G	22.22	LowQual;QDFilter	0.2	0,0,11,102	111	V91G	NS_CODING	ZNF419	ENST00000284020
chr19	58370657	C	A	202.91	QDFilter	0.95	61,153,0,0	212	Q250K	NS_CODING	ZNF587	ENST00000376209
chr3	75787794	A	G	42.2	LowCovFilter	21.1	0,0,2,0	2	I277T	NS_CODING	ZNF717	ENST00000478296
chr3	75790860	C	T	68.27	LowCovFilter	8.53	0,5,0,3	8	V22M	NS_CODING	ZNF717	ENST00000400845
chr3	75790953	C	T	68.27	LowCovFilter	22.76	0,0,0,3	3	NA	START_GAINED	ZNF717	ENST00000400845
chr19	56599405	C	G	76.7	LowCovFilter	10.96	0,4,3,0	6	G379A	NS_CODING	ZNF787	ENST00000270459
